# Efficacy of acupuncture in experimental intracerebral hemorrhage: a systematic review and meta-analysis

**DOI:** 10.3389/fneur.2024.1402129

**Published:** 2024-06-13

**Authors:** Zhe Wu, Mingyuan Jiao, Tianqi Wang, Baiwen Zhang, Hao Dong, Yunpeng Du, Jiayong Yao, Wei Zou

**Affiliations:** ^1^First Clinical Medical College, Heilongjiang University of Chinese Medicine, Harbin, China; ^2^Research and Teaching Department, Jinhua Maternal and Child Health Hospital, Jinhua, China; ^3^Integrated Chinese and Western Medicine Clinical Key Laboratory, Heilongjiang University of Chinese Medicine, Harbin, China; ^4^First Affiliated Hospital, Heilongjiang University of Chinese Medicine, Harbin, China

**Keywords:** intracerebral hemorrhage, acupuncture, meta-analysis, neurological function score, brain water content

## Abstract

**Objective:**

There is currently a lack of evidence in evidence-based medicine regarding acupuncture treatment for experimental intracerebral hemorrhage (ICH). The aim of this study was to systematically evaluate the efficacy of acupuncture treatment for experimental ICH based on neurological function scores and brain water content (BWC).

**Methods:**

Eight mainstream Chinese and English databases were searched. Outcome measures included neurological function scores and BWC, and subgroup analysis was conducted based on study characteristics.

**Results:**

A total of 32 studies were included. Meta-analysis results indicated that compared to the control group, the acupuncture group showed significant reductions in mNSS (MD = −3.16, *p* < 0.00001), Bederson score (MD = −0.99, *p* < 0.00001), Longa score (MD = −0.54, *p* < 0.0001), and brain water content (MD = −5.39, *p* < 0.00001). Subgroup analysis revealed that for mNSS, the autologous blood model (MD = −3.36) yielded better results than the collagenase model (MD = −0.92, *p* < 0.00001), and simple fixation (MD = −3.38) or no fixation (MD = −3.39) was superior to sham acupuncture (MD = −0.92, *p* < 0.00001). For BWC, the autologous blood model (MD = −7.73) outperformed the collagenase model (MD = −2.76, *p* < 0.00001), and GV20–GB7 (MD = −7.27) was more effective than other acupuncture points (MD = −2.92, *p* = 0.0006).

**Conclusion:**

Acupuncture significantly improves neurological deficits and brain edema in experimental ICH. Acupuncture at GV20 - GB7 is more effective than at other points. These findings support further studies to translate acupuncture into clinical treatment for human ICH.

**Systematic review registration:**

https://www.crd.york.ac.uk/prospero/, identifier CRD42023435584.

## 1 Introduction

Intracerebral hemorrhage (ICH) is the subtype of stroke with the highest mortality and disability rates worldwide, with an early mortality rate of up to 40%, significantly impacting human health (1). General therapies for ICH include early airway protection, urgent reversal of coagulation disorders, control of malignant hypertension, and surgical interventions (2). Additionally, stem cells, melatonin, and other agents have shown significant improvement in pathological indicators post-ICH (3, 4). Therefore, seeking effective therapies is crucial for achieving a favorable prognosis after ICH.

Acupuncture is recognized by the World Health Organization (WHO) as an effective treatment for stroke. Studies have shown that acute head acupuncture intervention can improve Clinical Neurological Function Deficit Scale scores in ICH patients (5). Furthermore, Li et al. reported that early head acupuncture intervention can improve the level of consciousness and serum brain-derived neurotrophic factor levels in ICH patients (6). Numerous preclinical studies on ICH have demonstrated that acupuncture can improve neurological function in ICH rats by modulating inflammatory factors, cell apoptosis, autophagy, and other mechanisms, thereby alleviating pathological changes in brain tissue (7–12). Therefore, a comprehensive analysis of the effectiveness of acupuncture in treating ICH is necessary.

Meta-analysis is a systematic statistical method aimed at combining and analyzing results from multiple independent studies to obtain conclusions with greater statistical significance and reliability. Two meta-analyses have shown that acupuncture therapy can effectively alleviate myofascial pain and pain caused by gastric cancer (13, 14). This article provides a systematic evaluation of the efficacy of acupuncture intervention in experimental ICH, offering insights for preclinical ICH research and serving as a reference for the selection of clinical ICH treatment methods.

## 2 Methods

This study was conducted based on the Preferred Reporting Items for Systematic Review and Meta-Analysis (PRISMA) 2020 Checklist, and detailed information can be found in Supplements 1 and 2. The study has been registered in PROSPERO with the registration number-CRD42023435584.

### 2.1 Criteria for inclusion and exclusion

We use authoritative neurological function scoring scales, including the modified Neurological Severity Score (mNSS), Bederson score, and Longa score, to observe the improvement of acupuncture on neurological function in ICH. Additionally, brain water content (BWC) is used to observe the extent of improvement in brain tissue edema in ICH.

The inclusion criteria are as follows:

1. Published animal experiments on ICH in either Chinese or English;
2. Intervention group receiving acupuncture or electroacupuncture treatment, control group receiving sham acupuncture or no treatment;
3. Outcome measures include mNSS, Bederson score, Longa score, or BWC.

The exclusion criteria are as follows:

1. Unable to extract valid outcome measures;
2. Duplicate publications;
3. Unable to access the full text;
4. Interventions combined with other therapies such as moxibustion, embedded thread at acupoints, etc.;
5. Literature not peer-reviewed, such as conference papers, master’s or doctoral theses, or monographs.

Two researchers (ZW and MJ) independently screened titles and abstracts from the search records, assessing potentially eligible studies for further evaluation of full texts. In cases of missing information, authors were contacted for clarification. Conflicts were resolved through discussion between the two authors.

### 2.2 Literature searches

The databases retrieved include PubMed, EMBASE, Cochrane Library, Web of Science, Chinese National Knowledge Infrastructure (CNKI), Wanfang Data Information Site, VIP Information Database, and Chinese Biomedical Literature Database (CBM). The retrieval period extends from the establishment of the databases to June 18, 2023. The retrieval strategy combines subject terms and free-text terms, and the search query is approximately as follows: (intracerebral hemorrhage) AND (acupuncture OR acupuncture therapy OR acupuncture points OR electroacupuncture) AND (rats OR mice OR models, animal OR animal experimentation). For a detailed retrieval strategy, please refer to Supplements 3.

### 2.3 Data selection and extraction

Two researchers (ZW and MJ) independently extracted data from the included studies and stored it in Excel. Any discrepancies between the two authors were resolved through discussion. Missing information was requested from the original authors via email. In cases of no response, this was noted in the discussion section.

The following information was extracted from the included literature:

1. First author and publication year;
2. Animal gender, species, weight, and sample size (n);
3. Type of anesthetic, injection method, and dosage;
4. Modeling method;
5. Intervention details, including selected acupoints, acupuncture methods, and timing;
6. Control measures;
7. Neurobehavioral and brain edema indicators (mean ± standard deviation).

The time point selected for extraction was day 3 after ICH. In cases of multiple duplicate articles reporting the same outcome data, we prioritized data from the article with higher research quality. If research quality was the same, we chose the more recent publication for data extraction. For experiments with multiple intervention groups, we extracted data from the group with the most favorable outcomes. If data were presented as mean ± standard error, we calculated mean ± standard deviation using the formula SD = SE × √n. When data were available only in image format, we used GetData Graph Digitizer 2.25 to extract values. Additionally, we conducted a thorough review of extracted data, excluding any unreliable data (such as data with excessively small standard deviations, given that neurofunctional scores are typically integers), to ensure accurate analysis results.”

### 2.4 Quality of bias assessment of included studies

The two researchers, ZW and MJ, independently used SYRCLE’s risk of bias tool to assess the quality of the included literature. SYRCLE, which stands for Systematic Review Center for Laboratory animal Experimentation, adapted this tool from the Cochrane risk of bias tool, and it is widely used to evaluate the methodological quality of animal experiments (15). The tool includes the following items:

1. Sequence generation;
2. Baseline characteristics;
3. Allocation concealment;
4. Random housing;
5. Blinding of animal caregivers and researchers;
6. Random outcome assessment;
7. Blinding of outcome assessors;
8. Incomplete outcome data;
9. Selective outcome reporting;
10. Other sources of bias.

The providers of the tool do not recommend evaluating a study as “high-quality” or “low-quality” based on a total score because summing scores inevitably involves assigning “weights” to specific domains in the tool, and it is difficult to prove that the assigned weights are reasonable (15). If there are disagreements during the quality assessment process, these are resolved through discussion or by involving a third researcher (WZ).

### 2.5 Data analysis

We conducted meta-analysis and subgroup analysis using Review Manager 5.3 and performed sensitivity analysis, meta-regression analysis, and publication bias assessment using Stata 14.0. Neurofunctional scores and BWC were treated as continuous variables, with Mean Difference (MD), 95% confidence intervals (CI), and *p*-values calculated to assess intergroup differences between intervention and control groups. Heterogeneity was evaluated using I^2^, with values exceeding 50% indicating high heterogeneity and prompting the use of a random-effects model; otherwise, a fixed-effects model was applied. Study results and heterogeneity assessment were presented using forest plots. Publication bias was assessed via funnel plots, and Egger’s test and the trim-and-fill method were employed for outcome measures with more than 10 studies to assess bias. Sensitivity analysis involved systematically excluding individual studies to observe their impact on the overall effect size. For outcomes with high heterogeneity, meta-regression analysis identified potential sources of heterogeneity. Subgroup analysis based on study characteristics such as animal species, ICH modeling methods, acupuncture points, and control measures was conducted for outcome measures with more than 10 studies. The Q-test based on variance analysis was used to assess subgroup differences, with a significance level of *p* < 0.05 considered statistically significant.

## 3 Results

### 3.1 Study selection

**Figure 1:**
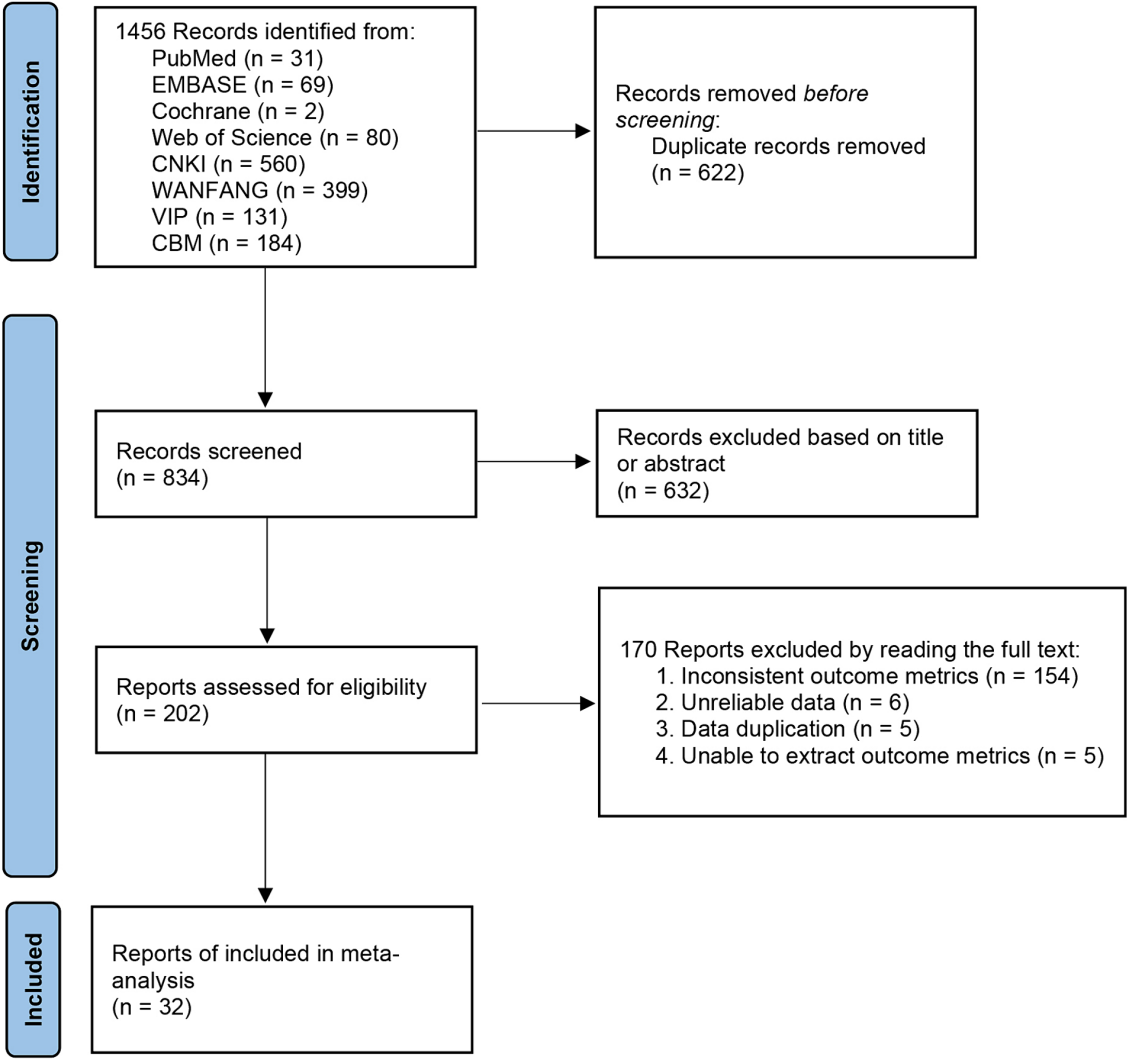
Flow chart of literature screening developed according to PRISMA guidelines.

Following the PRISMA guidelines, we conducted literature screening. A total of 1,456 articles were retrieved based on the search strategy. After removing duplicates, 834 articles remained. Upon reviewing titles and abstracts, 633 articles were excluded for the following reasons: (1) Not related to intracerebral hemorrhage; (2) Interventions involving non-acupuncture or combined with other therapies; (3) Human studies; (4) *In vitro* experiments; (5) Reviews, conference papers, patents, or books; (7) Theses. Among the 202 articles subjected to full-text assessment, 170 were excluded due to inconsistent outcome measures (*n* = 154), unreliable data (*n* = 6), data duplication (*n* = 5), and inability to extract outcome measures (*n* = 5). The remaining 32 studies were included in our meta-analysis (Figure 1).

### 3.2 Study characteristics

Among the 32 included studies (12, 16–46), 13 studies utilized Sprague–Dawley (SD) rats, 16 studies used Wistar rats, 2 studies employed guinea pigs, and 1 study utilized spontaneously hypertensive rats (SHR), all of which were male. Regarding anesthesia, 14 studies employed intraperitoneal injection of chloral hydrate, 12 studies used intraperitoneal injection of pentobarbital, and 6 studies did not specify the type of anesthetic used. In terms of modeling, 23 studies induced ICH by autologous blood injection into the caudate nucleus, while 9 studies induced caudate nucleus hemorrhage using collagenase. For the intervention group, 21 studies employed acupuncture from Baihui (GV20) to Qubin (GB7). In the control group, 16 studies underwent the same procedure for fixation but without acupuncture intervention as in the intervention group; 15 studies did not implement any measures, and 1 study used sham acupuncture as a control. Regarding outcome measures, 11 studies reported mNSS; 5 studies reported Bederson score; 6 studies reported Longa score; and 14 studies reported BWC. The assessment time ranged from 6 h to 28 days after ICH surgery, with all studies reporting outcomes on day 3. The detailed study characteristics of this research are provided in Table 1.

**Table 1:** The characteristics of the 32 included studies.

Author, year	Gender	Species, *n* = treatment/control	Weight	Anesthetic	Modeling method	Acupuncture method	Control measure	Neurobehavioral outcome	Histological outcome	Intergroup differences
Cao, 2022	Male	SD rats, 10/10	280 ± 20 g	2% pentobarbital (40 mg/kg), i.p.	Autologous blood, caudate nucleus	GV20 - GB7 (affected side); acupuncture 30 min/d after operation 12 h until sacrifice, rotating needles 5 min (190 ± 10 times/min), repeat thrice.	None	mNSS (6 h, 1d, 3d, 7d)		*p* < 0.01
Chen, 2022	Male	Wistar rats, 12/12	300 ± 20 g	1% pentobarbital (50 mg/kg), i.p.	Autologous blood, caudate nucleus	GV20, GV14; EA 30 min/d after operation until sacrifice, with the intensity of 2 mA and frequency of 2/15 Hz.	Fixed	mNSS (3d, 7d, 14d)		*p* < 0.01
Chen1, 2021	Male	Wistar rats, 6/6	300 ± 20 g	1% pentobarbital (50 mg/kg), i.p.	Autologous blood, caudate nucleus	GV20 - GB7 (affected side); acupuncture 30 min/d after operation 12 h until sacrifice, rotating needles 5 min (100 times/min), repeat thrice.	None	mNSS (1d, 3d, 7d)		*p* < 0.01
Chen2 2021	Male	1. Wistar rats, 12/12; 2. Wistar rats, 6/6	300 ± 20 g	1% pentobarbital (50 mg/kg), i.p.	Autologous blood, caudate nucleus	GV20 - GB7 (affected side); acupuncture 30 min/d after operation 12 h until sacrifice.	None	mNSS (1d, 3d, 7d)	BWC (1d, 3d, 7d)	1. *p* < 0.01; 2. *p* < 0.01
Han, 2021	Male	SD rats, 6/6	230–250 g	NR	Autologous blood, caudate nucleus	GV26, PC6 (bilateral); acupuncture 30 min/d after operation until sacrifice, twirling lifting and thrusting needles 1 min, repeat thrice.	Fixed	mNSS (6 h, 1d, 2d, 3d)		*p* > 0.05 (6 h, 1d, 2d), *p* < 0.05 (3d)
Jia, 2023	Male	Guinea pig, 12/12	300 ± 15 g	2% pentobarbital (40 mg/kg), i.p.	Autologous blood, caudate nucleus	GB20 (bilateral), TE17 (bilateral); EA 20 min/d after operation 12 h until sacrifice, with the intensity of 0.5 mA and frequency of 2 Hz.	Fixed	mNSS (1d, 3d, 7d, 14d)		*p* > 0.05 (1d), *p* < 0.01 (3d, 7d, 14d)
Liu1, 2018	Male	SD rats, 12/12	280–320 g	pentobarbital (60 mg/kg), i.p.	Autologous blood, caudate nucleus	GV20 - GB7 (affected side); acupuncture 30 min every 12 h after operation until sacrifice, rotating needles 5 min (200 times/min), repeat thrice.	Fixed	mNSS (6 h, 1d, 2d, 3d)		*p* < 0.05
Liu2, 2018	Male	1. SD rats, 24/24; 2. SD rats, 24/24	300 ± 20 g	10% Chloral hydrate (350 mg/kg), i.p.	Autologous blood, caudate nucleus	GV20 - GB7 (affected side); acupuncture 30 min every 12 h after operation until sacrifice, rotating needles 5 min (200 times/min), repeat thrice.	Fixed	mNSS (6 h, 12 h, 1d, 3d)	BWC (6 h, 12 h, 1d, 3d)	1. *p* < 0.05; 2. *p* > 0.05 (6 h, 12 h), *p* < 0.05 (1d, 3d)
Liu3, 2018	Male	1. SD rats, 6/6; 2. SD rats, 6/6	280–320 g	pentobarbital (60 mg/kg), i.p.	Autologous blood, caudate nucleus	GV20 - GB7 (affected side); acupuncture 30 min every 12 h after operation until sacrifice, rotating needles 5 min (200 times/min), repeat thrice.	Fixed	mNSS (6 h, 12 h, 1d, 3d, 7d)	BWC (6 h, 12 h, 1d, 3d)	1. *p* < 0.01; 2. *p* < 0.05(6 h), *p* < 0.01(12 h, 1d, 3d)
Xu, 2022	Male	SD rats, 18/18	260 ± 20 g	10% Chloral hydrate (350 mg/kg), i.p.	Autologous blood, caudate nucleus	GV20 - GB7 (affected side); acupuncture 30 min/d after operation until sacrifice, rotating needles 2 min (200 times/min), repeat thrice.	Fixed	mNSS (6 h, 3d, 7d)		*p* < 0.05
Zhu, 2017	Male	SD rats, 5/5	250–300 g	10% Chloral hydrate (0.5 mL/100 g), i.p.	Collagenase, caudate nucleus	GV20, GV14; EA 30 min/d after operation until sacrifice, with the intensity of 1 mA and frequency of 2 Hz.	Sham acupuncture	mNSS (1d, 3d, 5d, 7d, 14d)		*p* > 0.05 (1d, 3d), *p* < 0.01 (5d, 7d), *p* < 0.001 (14d)
Guan, 2017	Male	Wistar rats, 15/15	300 ± 20 g	10% Chloral hydrate (300 mg/kg), i.p.	Autologous blood, caudate nucleus	GV20 - GB7 (affected side); acupuncture 30 min/d after operation until sacrifice, rotating needles 2 min (200 times/min), repeat thrice.	Fixed	Bederson (6 h, 1d, 3d, 7d)		*p* > 0.05 (6 h), *p* < 0.05 (1d, 3d, 7d)
Kong, 2017	Male	SD rats, 6/6	300 ± 20 g	Chloral hydrate, i.p.	Autologous blood, caudate nucleus	GV20 - GB7 (affected side); acupuncture 30 min on 6 h, 1d, 3d, 7d after operation until sacrifice, rotating needles 3 min (200 times/min), repeat thrice.	None	Bederson (6 h, 1d, 3d, 7d)		*p* < 0.05 (6 h, 1d), *p* < 0.01 (3d, 7d)
Kuang, 2010	Male	Wistar rats, 10/10	350 ± 20 g	NR	Collagenase, caudate nucleus	GV20 - GB7 (affected side); acupuncture 30 min/d after operation until sacrifice, rotating needles 5 min (200 times/min), repeat thrice.	Fixed	Bederson (6 h, 1d, 2d, 3d, 7d)		*p* > 0.05 (6 h, 1d), *p* < 0.01 (2d, 3d, 7d)
Li, 2021	Male	1. SD rats, 6/6; 2. SD rats, 6/6	NR	pentobarbital (50 mg/kg), i.p.	Autologous blood, caudate nucleus	GV20 - GB7; acupuncture 30 min/d after operation 1 h until sacrifice.	None	Bederson (3d)	BWC (3d)	*p* < 0.05
Xiao, 2019	Male	Wistar rats, 12/12	280–320 g	10% Chloral hydrate, i.p.	Autologous blood, caudate nucleus	GV20 - GB7 (affected side); acupuncture 30 min/d after operation until sacrifice, rotating needles qucikly, repeat thrice.	None	Bederson (6 h, 12 h, 1d, 3d, 7d)		*p* > 0.05 (6 h, 12 h), *p* < 0.01 (1d, 3d, 7d)
Chen, 2018	Male	Wistar rats, 6/6	350 ± 20 g	10% Chloral hydrate (350 mg/kg), i.p.	Autologous blood, caudate nucleus	GV20 - GB7 (affected side); acupuncture 30 min/d after operation until sacrifice, rotating needles 5 min (100 times/min), repeat thrice.	None	Longa (1d, 3d, 7d)		*p* < 0.01
Chen, 2019	Male	Wistar rats, 6/6	350 ± 20 g	10% Chloral hydrate (350 mg/kg), i.p.	Autologous blood, caudate nucleus	GV20 - GB7 (affected side); acupuncture 30 min/d after operation 12 h until sacrifice.	None	Longa (1d, 3d, 7d)		*p* < 0.01
Chen, 2020	Male	Wistar rats, 6/6	350 ± 20 g	10% Chloral hydrate (350 mg/kg), i.p.	Autologous blood, caudate nucleus	GV20 - GB7 (affected side); acupuncture 30 min/d after operation 1d until sacrifice.	Fixed	Longa (1d, 3d, 7d)		*p* > 0.05 (1d), *p* < 0.05 (3d, 7d)
Jia, 2020	Male	Guinea pig, 8/8	300 ± 15 g	2% pentobarbital (40 mg/kg), i.p.	Autologous blood, caudate nucleus	GB20 (bilateral), TE17 (bilateral); EA 20 min/d after operation 12 h until sacrifice, with the intensity of 0.5 mA and frequency of 2 Hz.	Fixed	Longa (1d, 3d, 7d, 14d)		*p* > 0.05 (1d), *p* < 0.01 (3d, 14d), *p* < 0.05 (7d)
Wang, 2016	Male	SD rats, 12/12	250–300 g	10% Chloral hydrate (400 mg/kg), i.p.	Collagenase, caudate nucleus	GV20 - GB7 (affected side), EA 30 min/d after operation 6 h until sacrifice, with the intensity of 0.2 mA and frequency of 2 Hz.	Fixed	Longa (6 h, 1d, 3d, 7d)		*p* > 0.05 (6 h), *p* < 0.05 (1d, 3d, 7d)
Yu, 2019	Male	Wistar rats, 12/12	340 ± 30 g	pentobarbital (60 mg/kg), i.p.	Autologous blood, caudate nucleus	GV20 - GB7 (affected side), acupuncture 30 min/d after operation until sacrifice, rotating needles 5 min (200 times/min), repeat twice.	None	Longa (3d, 7d, 14d, 21d, 28d)		*p* > 0.05 (3d, 7d), *p* < 0.05 (14d, 21d, 28d)
Cai, 2020	Male	SD rats, 9/9	300 ± 15 g	pentobarbital (40 mg/kg)	Autologous blood, caudate nucleus	GB20 (left), TE17 (right); EA 30 min/d after operation until sacrifice.	Fixed		BWC (3d)	*p* < 0.05
Dai, 2002	Male	SD rats, 7/8	250 ± 15 g	2% pentobarbital (40 mg/kg), i.p.	Collagenase, caudate nucleus	GV16, GV15; EA 30 min/d after operation 2 h until sacrifice.	Fixed		BWC (3d)	*p* < 0.05
Li, 2015	Male	Wistar rats, 4/4	250–300 g	NR	Collagenase, caudate nucleus	GV20-GB5 (affected side); acupuncture 30 min/d after operation 6 h until sacrifice, rotating needles 1 min (200 times/min), repeat thrice.	None		BWC (12 h, 1d, 3d, 7d)	*p* < 0.05
Shu, 2011	Male	SHR, 3/3	250 ± 20 g	NR	Collagenase, caudate nucleus	GV26; EA 30 min/d after operation 6 h until sacrifice, with the intensity of 1 mA and frequency of 120/min.	None		BWC (6 h, 1d, 3d, 7d)	*p* < 0.01
Sun, 2017	Male	Wistar rats, 4/4	350.21 ± 20.33 g	NR	Autologous blood, caudate nucleus	GV20 - GB7; acupuncture 30 min/d after operation until sacrifice, rotating needles 5 min (200 times/min), repeat thrice.	None		BWC (6 h, 1d, 2d, 3d, 7d)	*p* > 0.05 (6 h), *p* < 0.05 (1d, 2d, 3d, 7d)
Wang, 2012	Male	Wistar rats, 6/6	200 g	NR	Collagenase, caudate nucleus	GV20, GV26, ST36; EA 30 min/d after operation 3 h, 1d, 2d, 3d until sacrifice, with the intensity of 1 mA and frequency of 2 Hz.	None		BWC (6 h, 1d, 2d, 3d)	*p* < 0.05
Wang2, 2012	Male	SD rats, 8/8	250–280 g	10% Chloral hydrate, i.p.	Collagenase, caudate nucleus	GV20 - GB7 (affected side); acupuncture 30 min/d after operation until sacrifice, rotating needles 5 min (200 times/min), repeat twice.	None		BWC (3d)	*p* < 0.05
Xu, 2017	Male	Wistar rats, 8/8	250–300 g	10% Chloral hydrate (400 mg/kg), i.p.	Collagenase, caudate nucleus	GV20, EX-HN5 (affected side); EA 30 min/d after operation 6 h, 1d until sacrifice.	None		BWC (6 h, 1d, 3d, 7d)	*p* > 0.05 (6 h, 1d), *p* < 0.05 (3d, 7d)
Zou, 2010	Male	Wistar rats, 10/10	350 ± 20 g	10% Chloral hydrate (350 mg/kg), i.p.	Autologous blood, caudate nucleus	GV20 - GB7 (affected side); acupuncture 30 min/d after operation until sacrifice, rotating needles 5 min (200 times/min), repeat thrice.	Fixed		BWC (6 h, 1d, 2d, 3d, 7d)	*p* > 0.05 (6 h), *p* < 0.01 (1d, 2d, 3d, 7d)
Zou, 2011	Male	Wistar rats, 6/6	350 ± 20 g	10% Chloral hydrate (350 mg/kg), i.p.	Autologous blood, caudate nucleus	GV20 - GB7 (affected side); acupuncture 30 min/d after operation until sacrifice, rotating needles 2 min (200 times/min), repeat thrice.	Fixed		BWC (6 h, 1d, 2d, 3d, 7d)	*p* > 0.05 (6 h), *p* < 0.01 (1d, 2d, 3d, 7d)

SD, Sprague–Dawley; SHR, spontaneously hypertensive rats; i.p., intraperitoneal injection; NR, not report; EA, electroacupuncture; mNSS, modified neurological severity score; BWC, brain water content.

### 3.3 Quality of bias of included studies

As shown in Table 2, among the 32 studies, for selection bias, 28 studies (87.5%) used randomized group allocation, while only one study (3.13%) reported balanced baseline characteristics between the intervention and control groups. None of the studies clearly described allocation concealment. For performance bias, 15 studies (46.88%) reported randomized housing of animals, but none mentioned whether blinding was implemented for caregivers and researchers performing acupuncture. For detection bias, three studies (9.38%) conducted randomized outcome assessments, and only one study (3.13%) reported blinding of assessors. For attrition bias, all studies reported complete study data. Regarding reporting bias, 31 studies (96.88%) did not selectively report results. For other biases, 29 studies (90.63%) were judged to have no other risks of bias.

**Table 2:** Methodological quality of the 32 included studies.

Study	(1)	(2)	(3)	(4)	(5)	(6)	(7)	(8)	(9)	(10)
Cao, 2022	U	U	U	Y	U	N	U	Y	Y	Y
Chen, 2022	Y	U	U	Y	U	N	U	Y	Y	U
Chen1, 2021	Y	U	U	Y	U	N	U	Y	Y	Y
Chen2, 2021	Y	U	U	Y	U	N	U	Y	Y	Y
Han, 2021	U	U	U	Y	U	N	U	Y	Y	Y
Jia, 2023	Y	U	U	Y	U	Y	U	Y	Y	Y
Liu1, 2018	U	U	U	U	U	N	U	Y	Y	Y
Liu2, 2018	U	U	U	U	U	N	U	Y	Y	Y
Liu3, 2018	U	U	U	Y	U	N	U	Y	Y	Y
Xu, 2022	U	U	U	Y	U	N	U	Y	Y	Y
Zhu, 2017	U	U	U	Y	U	N	Y	Y	Y	Y
Guan, 2017	U	U	U	Y	U	N	U	Y	Y	Y
Kong, 2017	U	U	U	U	U	N	U	Y	Y	U
Kuang, 2010	U	U	U	U	U	N	U	Y	Y	Y
Li, 2021	N	U	U	U	U	N	U	Y	Y	Y
Xiao, 2019	Y	U	U	Y	U	N	U	Y	Y	U
Chen, 2018	U	U	U	U	U	N	U	Y	Y	Y
Chen, 2019	U	U	U	U	U	N	U	Y	Y	Y
Chen, 2020	U	U	U	U	U	N	U	Y	Y	Y
Jia, 2020	Y	U	U	Y	U	N	U	Y	Y	Y
Wang, 2016	U	U	U	U	U	N	U	Y	Y	Y
Yu, 2019	U	U	U	U	U	N	U	Y	Y	Y
Cai, 2020	U	U	U	Y	U	U	U	Y	Y	Y
Dai, 2002	N	U	U	U	U	N	U	Y	Y	Y
Li, 2015	U	U	U	U	U	N	U	Y	Y	Y
Shu, 2011	N	U	U	U	U	N	U	Y	Y	Y
Sun, 2017	U	Y	U	U	U	U	U	Y	Y	Y
Wang, 2012	U	U	U	U	U	N	U	Y	Y	Y
Wang2, 2012	N	U	U	U	U	N	U	Y	Y	Y
Xu, 2017	U	U	U	Y	U	N	U	Y	N	Y
Zou, 2010	U	U	U	U	U	N	U	Y	Y	Y
Zou, 2011	U	U	U	Y	U	N	U	Y	Y	Y

(1) Sequence generation; (2) Baseline characteristics; (3) Allocation concealment; (4) Random housing; (5) blinding of trial caregivers and researchers; (6) Random outcome assessment; (7) Blinding of outcome assessors; (8) Incomplete outcome data; (9) Selective outcome reporting; (10) Other sources of bias; Y, low risk of bias; N, high risk of bias; and U, unclear risk of bias.

### 3.4 Intervention effects

**Figure 2:**
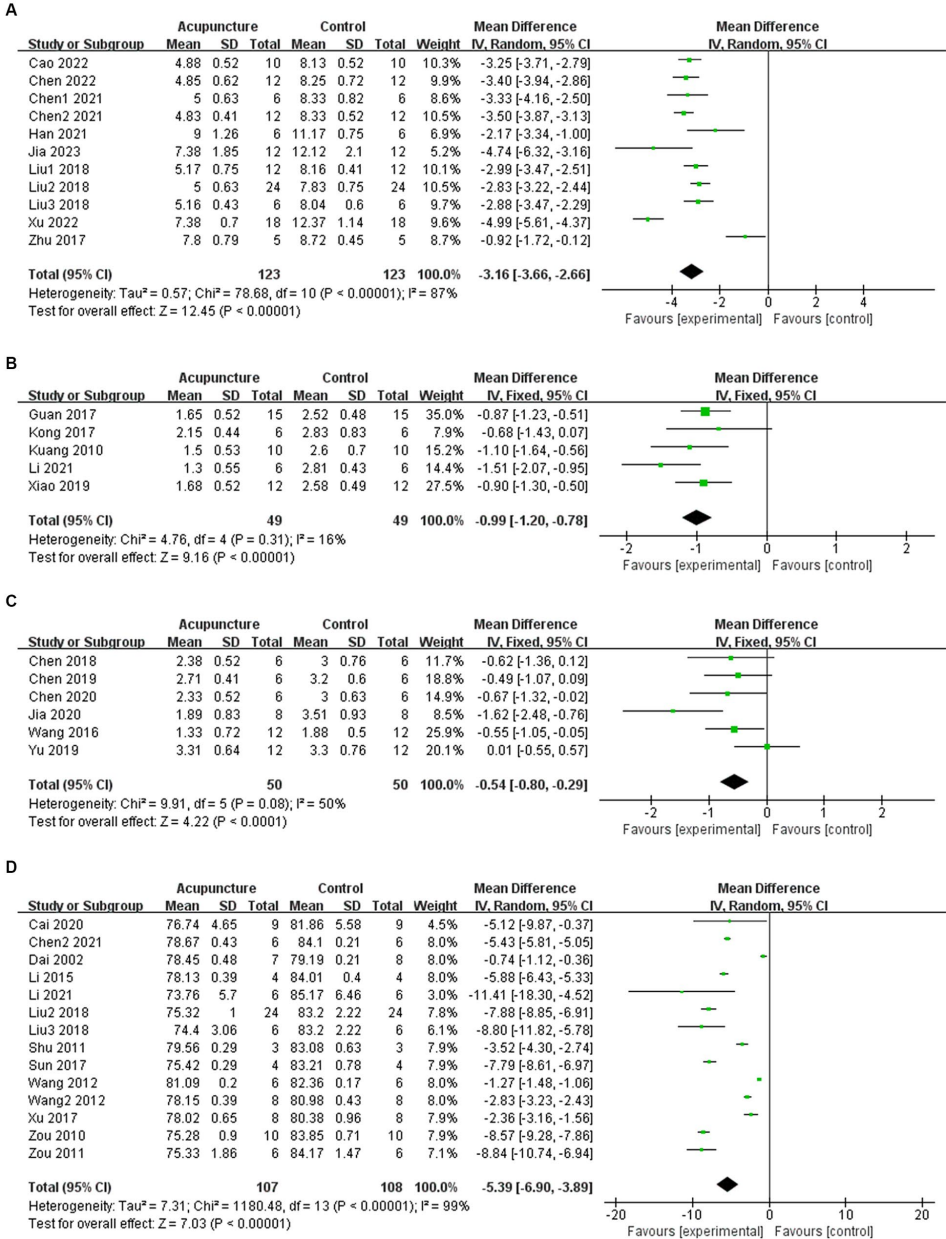
Summary statistics of effect sizes for **(A)** modified neurological severity score, **(B)** Bederson score, **(C)** Longa score, and **(D)** brain water content.

In 11 studies, acupuncture demonstrated a significant improvement in mNSS after experimental ICH (*n* = 246, MD = −3.16, 95% CI [−3.66 ~ −2.66], *p <* 0.00001; heterogeneity Chi^2^ = 78.68, df = 10 (*p* < 0.00001), I^2^ = 87%; Figure 2A). Among the 5 studies examining Bederson score, acupuncture also showed a significant effect in improving outcomes after experimental ICH (*n* = 98, MD = −0.99, 95% CI [−1.20 ~ −0.78], *p <* 0.00001; heterogeneity Chi^2^ = 4.76, df = 4 (*p* = 0.31), I^2^ = 16%; Figure 2B). Additionally, in 6 studies, acupuncture led to a significant improvement in Longa score after experimental ICH (*n* = 100, MD = −0.54, 95% CI [−0.80 ~ −0.29], *p <* 0.0001; heterogeneity Chi^2^ = 9.91, df = 5 (*p* = 0.08), I^2^ = 50%; Figure 2C). For BWC, 14 studies demonstrated a significant improvement following acupuncture treatment after experimental ICH (*n* = 215, MD = −5.39, 95% CI [−6.90 ~ −3.89], *p <* 0.00001; heterogeneity Chi^2^ = 1180.48, df = 13 (*p <* 0.00001), I^2^ = 99%; Figure 2D). The heterogeneity in mNSS and BWC outcomes is statistically significant. Therefore, meta-regression and subgroup analysis were conducted to explore the sources of heterogeneity.

### 3.5 Sensitivity analysis

**Figure 3:**
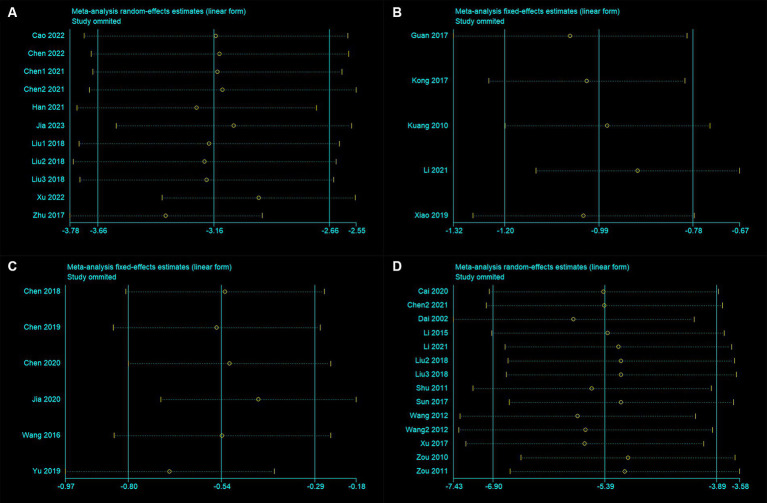
Sensitivity analysis for **(A)** modified neurological severity score, **(B)** Bederson score, **(C)** Longa score, and **(D)** brain water content.

The sensitivity analysis results indicate that after excluding any individual trial, there were no significant changes in the results for mNSS, Bederson, Longa, and BWC (Figures 3A–D). This indicates good stability of the meta-analysis results. For both mNSS and BWC outcomes, removing any single study did not lead to significant changes in heterogeneity, indicating that the source of heterogeneity is not attributed to any specific study.

### 3.6 Publication bias

**Figure 4:**
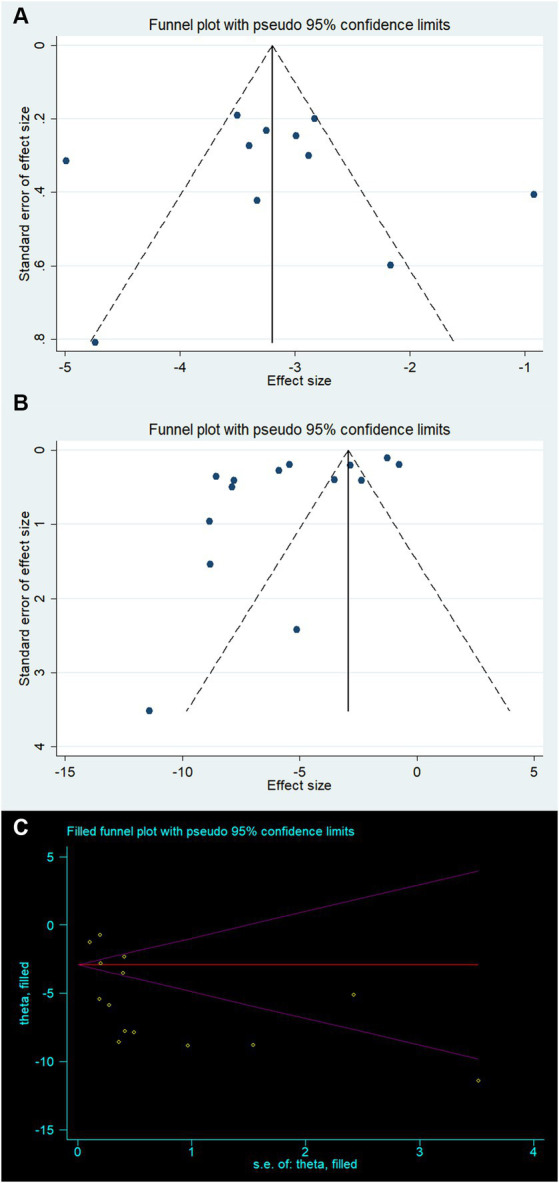
Publication bias for **(A)** modified neurological severity score, **(B)** brain water content, and **(C)** trim-and-fill method to brain water content.

Creating a funnel plot is not recommended due to the limited number of studies for both Bederson and Longa (both less than 10). Instead, Egger’s test was employed to observe potential publication bias. The funnel plot for mNSS appears to be relatively symmetrical, while the funnel plot for BWC shows some asymmetry (Figures 4A,B). Egger’s test results confirmed the absence of significant publication bias in mNSS, Bederson, and Longa outcomes, but indicated significant bias in BWC (mNSS *p* = 0.846, Bederson *p* = 0.67, Longa *p* = 0.12, BWC *p* = 0.026). For BWC, we utilized the trim-and-fill method to estimate the number of potentially missing studies and observed changes in the combined effect size estimate. The effect size estimate after correction remained consistent with the uncorrected value (MD −5.39, 95% CI [−6.90 ~ −3.89], *p* < 0.001), suggesting that there are no “missing” studies (Figure 4C).

### 3.7 Meta-regression

Due to the observed high heterogeneity in the analysis results for mNSS and BWC (with I^2^ values of 87 and 99% respectively), we conducted meta-regression analysis to examine the correlation between study characteristics and intervention effects, aiming to identify the sources of this heterogeneity. The results indicate that: (1) for mNSS, the modeling method is a significant contributor to its heterogeneity (*p* = 0.018); (2) for BWC, acupuncture points (*p* = 0.005) and modeling method (*p* < 0.001) are significant contributors to its heterogeneity.

### 3.8 Subgroup analysis

Subgroup analysis was conducted based on study characteristics (species, modeling method, acupuncture points, or control measures) to explore the sources of heterogeneity for mNSS and BWC. However, since some studies did not specify the name of the anesthetic used, subgroup analysis was not performed on this factor. (1) The subgroup analysis results for mNSS showed significant differences in the estimated effects among different modeling methods (Chi^2^ = 28.46, df = 1, *p* < 0.00001). The autologous blood model (MD = −3.36, 95% CI [−3.78 ~ −2.95], *p* < 0.00001) yielded better results compared to the collagenase model (MD = −0.92, 95% CI [−1.72 ~ −0.12], *p* = 0.02). There were also significant differences among different control measures (Chi^2^ = 33.55, df = 2, *p* < 0.00001). The effects estimated for simple fixation (MD = −3.38, 95% CI [−4.02 ~ −2.73], *p* < 0.00001) or no intervention (MD = −3.39, 95% CI [−3.66 ~ −3.12], *p* < 0.00001) were larger, while the effect estimated for sham acupuncture (MD = −0.92, 95% CI [−1.72 ~ −0.12], *p* = 0.02) was the smallest. In addition, there were no significant differences observed between different species or acupuncture points. (2) The subgroup analysis for BWC demonstrated a significant difference in the estimated effects among different acupuncture points (Chi^2^ = 11.77, df = 1, *p* = 0.0006). GV20 - GB7 (MD = −7.27, 95% CI [−9.13 ~ −5.40], *p* < 0.00001) showed better results compared to other acupuncture points (MD = −2.92, 95% CI [−4.56 ~ −1.28], *p* = 0.0005). There were also significant differences among different modeling methods (Chi^2^ = 24.25, df = 1, *p* < 0.00001). The effects estimated for the autologous blood model (MD = −7.73, 95% CI [−9.15 ~ −6.31], *p* < 0.00001) were larger compared to the collagenase model (MD = −2.76, 95% CI [−4.13 ~ −1.38], *p* < 0.0001). Additionally, there were no significant differences observed between different species or control measures. The detailed information for subgroup analysis is available in Tables 3, 4.

**Table 3:** Subgroup analysis for modified neurological severity score.

Subgroups	No. of studies	MD [95%CI]	*p*	Heterogeneity test			*p* (between groups)
				Q	I^2^	*p*	
Species							*p* = 0.10
SD	7	−2.90 [−3.64, −2.17]	*p* < 0.00001	69.78	91%	*p* < 0.00001	
Wistar	3	−3.45 [−3.74, −3.16]	*p* < 0.00001	0.18	0%	*p* = 0.91	
Guinea pigs	1	−4.74 [−6.32, −3.16]	*p* < 0.00001	N/A	N/A	N/A	
Methods of ICH							*p* < 0.00001
Autoblood	10	−3.36 [−3.78, −2.95]	*p* < 0.00001	45.79	80%	*p* < 0.00001	
Collagenase	1	−0.92 [−1.72, −0.12]	*p* = 0.02	N/A	N/A	N/A	
Acupoints							*p* = 0.43
GV20 – GB7	7	−3.38 [−3.86, −2.90]	*p* < 0.00001	38.92	85%	*p* < 0.00001	
Others	4	−2.74 [−4.25, −1.22]	*p* = 0.0004	32.84	91%	*p* < 0.00001	
Methods of control groups							*p* < 0.00001
Fixed	7	−3.38 [−4.02, −2.73]	*p* < 0.00001	74.94	91%	*p* < 0.00001	
Sham	1	−0.92 [−1.72, −0.12]	*p* = 0.02	N/A	N/A	N/A	
None	3	−3.39 [−3.66, −3.12]	*p* < 0.00001	0.71	0%	*p* = 0.70	

SD, Sprague–Dawley; MD, Mean Difference; CI, confidence intervals.

**Table 4:** Subgroup analysis for brain water content.

Subgroups	No. of studies	MD [95%CI]	*p*	Heterogeneity test			*p* (between groups)
				Q	I^2^	*p*	
Species							*p* = 0.11
SD	6	−5.34 [−7.74, −2.94]	*p* < 0.0001	221.81	98%	*p* < 0.00001	
Wistar	7	−5.70 [−8.11, −3.28]	*p* < 0.00001	925.69	99%	*p* < 0.00001	
SHR	1	−3.52 [−4.30, −2.74]	*p* < 0.00001	N/A	N/A	N/A	
Methods of ICH							*p*<0.00001
Autoblood	8	−7.73 [−9.15, −6.31]	*p* < 0.00001	89.84	92%	*p* < 0.00001	
Collagenase	6	−2.76 [−4.13, −1.38]	*p* < 0.0001	310.75	98%	*p* < 0.00001	
Acupoints							*p* = 0.0006
GV20 – GB7	8	−7.27 [−9.13, −5.40]	*p* < 0.00001	310.94	98%	*p* < 0.00001	
Others	6	−2.92 [−4.56, −1.28]	*p* = 0.0005	288.91	98%	*p* < 0.00001	
Methods of control groups							*p* = 0.34
Fixed	6	−6.66 [−10.79, −2.53]	*p* < 0.00001	512.83	99%	*p* < 0.00001	
None	8	−4.47 [−6.22, −2.72]	*p* < 0.00001	660.58	99%	*p* < 0.00001	

SD, Sprague–Dawley; SHR, spontaneously hypertensive rats; MD, Mean Difference; CI, confidence intervals.

## 4 Discussion

### 4.1 Summary of evidence

A previous meta-analysis reviewed studies up to 2009 and found that scalp acupuncture may improve neurological deficits in patients with acute hypertensive ICH (47). Additionally, Li et al. reviewed studies up to 2013 and demonstrated potential efficacy of GV20-based scalp acupuncture in acute ICH animal models (48). However, these studies only included literature on acupuncture points located on the head and are outdated, necessitating updated and comprehensive data to support their conclusions. A meta-analysis by Wang et al. indicated that acupuncture combined with minimally invasive surgery or basic treatment has certain efficacy for hypertensive intracerebral hemorrhage compared to minimally invasive surgery or basic treatment alone (49). Unfortunately, this analysis did not perform subgroup analyses based on study characteristics to discuss the differences between various acupuncture methods or different populations. Therefore, we screened all studies on acupuncture treatment for ICH to date and conducted subgroup analyses to explore the potential impact of different study characteristics on effect sizes. The results showed that acupuncture intervention significantly improved neurobehavioral and brain edema outcomes in experimental ICH, including mNSS (MD = −3.16, *p* < 0.00001), Bederson score (MD = −0.99, *p* < 0.00001), Longa score (MD = −0.54, *p* < 0.0001), and BWC (MD = −5.39, *p* < 0.00001). Subgroup analyses indicated that different modeling methods, acupuncture points, or control measures may influence effect sizes.

### 4.2 Potential mechanisms of acupuncture on intracerebral hemorrhage

Current research indicates that acupuncture therapy can reduce inflammatory responses and improve synaptic plasticity (50, 51). Additionally, numerous studies have explored the precise mechanisms of acupuncture in treating ICH. Research has shown that acupuncture promotes microglial M2 polarization through the miR-34a-5p/Klf4 and PD-1/PD-L1 signaling pathways, resulting in anti-inflammatory effects (29, 52). Besides its anti-inflammatory properties, acupuncture enhances mitochondrial autophagy after ICH, reduces the number of apoptotic cells, and inhibits ferroptosis in neurons (7–12). Furthermore, acupuncture improves the integrity of the blood–brain barrier by downregulating the expression of caveolin-1 and matrix metalloproteinases (53). Additionally, acupuncture promotes the secretion of neurotrophic factors and angiogenesis around the hematoma following ICH (6, 54, 55). Based on these mechanisms, acupuncture exerts neuroprotective effects, thereby improving neurological recovery after ICH.

### 4.3 Methodological quality

Quality assessment results indicate that all studies reported complete data, with the vast majority having no selective reporting or other risks of bias. In terms of experimental design, most studies implemented randomized group allocation, and nearly half conducted randomized housing of animals. However, the degree of randomization in outcome assessment was low. Additionally, blinding was poorly implemented throughout the experimental process. To some extent, blinding is more challenging for acupuncture compared to other treatments, as those performing acupuncture are aware of whether they are administering the treatment (unlike medical treatments, which can use placebos that look similar). Nonetheless, randomization and blinding are essential for high-quality animal experiments (56). Future studies could address the blinding issue by employing acupuncturists who are unaware of the experimental design.

### 4.4 Interpretation of subgroup analysis by species

Animal studies of ICH typically involve the use of SD rats and Wistar rats, with some studies also utilizing SHR rats and guinea pigs. Kunze et al. reported that SD rats and Wistar rats show similar modified Bederson test scores at several hours or days after middle cerebral artery occlusion (57), indicating that different species exhibit similar neurological deficits post-stroke. Our study results demonstrate no significant differences in mNSS scores among SD rats, Wistar rats, and guinea pigs. Likewise, there were no significant differences in BWC among SD rats, Wistar rats, and SHR rats. This indicates that acupuncture intervention has consistent effects across different species following ICH.

### 4.5 Interpretation of subgroup analysis by methods of ICH induction

The most widely used methods for inducing ICH include autologous blood injection and collagenase injection, with other methods such as microballoon inflation and spontaneous intracerebral hemorrhage. Zhao et al. reported that in SD rat models of ICH induced by collagenase or non-heparinized autologous blood, there were no statistically significant differences between the two groups in Bederson score and Longa score on 2 day, as well as BWC on 4 day postoperatively (58). However, a study on mice ICH indicated that the BWC on 3 day in the collagenase-induced ICH model was significantly higher than in the autologous blood-induced model, and the disruption of the blood–brain barrier was more severe in the collagenase group (59). Additionally, bacterial collagenase may increase inflammatory responses and have neurotoxic effects, particularly at high doses (60). Our results indicate that the improvement in mNSS and BWC after acupuncture intervention is higher in the autologous blood model compared to the collagenase model, which may be related to the additional adverse effects of collagenase on brain tissue. Intra-group heterogeneity remains significant, which may be related to the dosage of autologous blood and collagenase used. Therefore, a standardized modeling method is crucial for reducing heterogeneity and providing sufficient evidence.

### 4.6 Interpretation of subgroup analysis by acupoints

Baihui (GV20) is an empirically effective acupuncture point for stroke treatment, situated 7 cun above the midpoint of the anterior hairline, at the intersection point of a line connecting the tips of both ears and the midline of the head. Qubin (GB7) is located at the intersection of the vertical line at the anterior temporal hairline and the horizontal line through the tip of the ear. In most pre-clinical studies of ICH, GV20 was consistently selected, and needling was directed toward GB7, often employing rapid twisting and stimulating techniques. Numerous studies have reported that acupuncture at GV20 - GB7 alleviates early neurological damage after experimental ICH through mechanisms such as regulating cell apoptosis, inflammation, ferroptosis, microglial polarization, and mitochondrial autophagy, thereby promoting brain function recovery (7, 11, 24, 29, 61–63). Our study indicates that compared to other acupoints, acupuncture at GV20 - GB7 has a better effect on improving BWC after ICH, but there is no significant difference in mNSS. Considering the potential mechanisms of scalp acupuncture, we argue that GV20 - GB7 is the most effective acupoint for acupuncture treatment of ICH.

### 4.7 Interpretation of subgroup analysis by control measures

In 1995, the WHO defined sham acupuncture in their “Clinical Guidelines on Acupuncture” as a form of acupuncture based on acupuncture theory but deemed inappropriate for treating the specific disease. Since then, Sham acupuncture has been widely used in clinical acupuncture randomized controlled trials. Presently, sham acupuncture encompasses superficial needling or non-penetration of acupuncture points, as well as needling or non-penetration at non-acupuncture points. However, some of these methods may not fully account for the intricate mechanisms of acupuncture, as shallow needling at or near acupuncture points might also have therapeutic effects on the disease (64). Dincer argues that categorizing all these varied measures as “sham acupuncture” controls is misleading and scientifically unacceptable (65). In 2022, the WHO’s “International Standard Terminologies on Traditional Medicine” defines sham acupuncture as a “control that simulates acupuncture needling without actual penetration, ideally producing no physiological response,” avoiding skin penetration effects. Our results indicate that for BWC, there were no significant differences observed between the control group with simple fixation, taking no action, and sham acupuncture. However, for mNSS, simple fixation and taking no action yielded better results compared to sham acupuncture. This discrepancy may stem from the small sample size in the sham acupuncture subgroup, resulting in unstable outcomes, or it may be due to the use of a skin-penetrating sham acupuncture method with the connection of an electroacupuncture device in the control group, inducing a partial “treatment effect.” Sham acupuncture primarily functions as a placebo to blind the subjects, preventing them from knowing whether they are receiving treatment. This challenge is not applicable in animal experiments. In the future, exploring a scientifically sound sham acupuncture method that can blind both the practitioner and the subjects while minimizing additional effects on the disease is essential.

### 4.8 Interpretation of heterogeneity

Meta-regression analysis revealed that the modeling method significantly contributed to the heterogeneity in mNSS, while both acupuncture points and modeling methods were significant sources of heterogeneity for BWC. We conducted subgroup analyses based on predefined study characteristics to pinpoint sources of heterogeneity. However, persistent high heterogeneity within certain subgroups remains, possibly due to the interaction effects among different study characteristics, such as modeling methods and acupuncture points. To some extent, heterogeneity is inherent in meta-analyses of animal experiments because different experimental environments or methods can be important sources of heterogeneity. For example, the site of brain water content measurement might be the basal ganglia, the hemorrhagic hemisphere, or the whole brain. However, some articles did not report the measurement site, preventing further analysis. The random effects model can tolerate a certain degree of heterogeneity and provide a conservative estimate of effect sizes. We used this model to analyze mNSS and BWC. Additionally, the fixed effects model was applied to the Bederson and Longa scores, which had low heterogeneity. The results indicate that acupuncture can significantly improve neurological deficits and brain edema in experimental ICH. Nonetheless, future studies should focus on standardizing experimental procedures and providing detailed reports of experimental steps to enhance the stability and reproducibility of the results.

### 4.9 Strengths and limitations

This study has undertaken significant efforts. Firstly, it has reviewed all existing literature on acupuncture treatment for experimental ICH, whereas the last similar review was conducted a decade ago. Secondly, during data extraction, we excluded studies with unreliable data (e.g., studies with unreasonably small standard deviations in neurological function scores, given that all four scales use integer scores). Additionally, due to the high heterogeneity of two indicators, we employed meta-regression and subgroup analysis to identify sources of heterogeneity and to explore the potential effects of different species, modeling methods, acupuncture points, and control measures on effect sizes. Finally, sensitivity analysis and publication bias tests were conducted to confirm the stability of the results.

This study still has some limitations. Firstly, meta-regression results indicated that the modeling method and acupuncture points were significant sources of heterogeneity. However, high heterogeneity remained within subgroups after subgroup analysis. We used a random effects model to conservatively estimate the impact of acupuncture on the mNSS and BWC indicators of experimental ICH. Despite this, the results should be interpreted with caution. Additionally, current literature provides limited information on the injection doses of autologous blood and collagenase during modeling, which directly affects the extent of brain tissue damage post-hemorrhage. Furthermore, the literature often fails to report the specific brain regions where BWC was measured, affecting the stability of BWC effect size estimates. Therefore, future research should aim to thoroughly report experimental designs to minimize heterogeneity across studies, striving for robust evidence-based results to guide the clinical translation of acupuncture treatment for ICH.

## 5 Conclusion

Acupuncture significantly improves neurological deficits and brain edema in experimental ICH. Acupuncture at GV20 - GB7 is more effective than at other points. These findings support further studies to translate acupuncture into clinical treatment for human ICH.

## Data availability statement

The original contributions presented in the study are included in the article/Supplementary material, further inquiries can be directed to the corresponding author.

## Author contributions

ZW: Conceptualization, Formal analysis, Investigation, Methodology, Software, Visualization, Writing – original draft. MJ: Formal analysis, Software, Validation, Visualization, Writing – original draft. TW: Methodology, Writing – review & editing. BZ: Validation, Writing – review & editing. HD: Writing – review & editing. YD: Validation, Writing – review & editing. JY: Writing – review & editing. WZ: Funding acquisition, Project administration, Supervision, Writing – review & editing.
