# Supplementary material for: Efficacy of acupuncture in experimental intracerebral hemorrhage: a systematic review and meta-analysis
Source: Front Neurol. 2024 Jun 13;15:1402129. doi: 10.3389/fneur.2024.1402129 (PMC11208702; doi:10.3389/fneur.2024.1402129)
Supplement: Supplementary file 1 [file Table_1.docx]

### Supplement 1. PRISMA 2020 Checklist

| **Section and Topic** | **Item #** | **Checklist item** | **Location where item is reported** |
| --- | --- | --- | --- |
| **TITLE** | | |  |
| Title | 1 | Systematic Review of Acupuncture Intervention in Experimental Intracerebral Hemorrhage. |  |
| **ABSTRACT** | | |  |
| Abstract | 2 | See the PRISMA 2020 for Abstracts checklist. |  |
| **INTRODUCTION** | | |  |
| Rationale | 3 | Numerous studies have shown that acupuncture improves the neurological function of rats in the acute phase of ICH by regulating inflammatory factors, programmed cell death, autophagy, and other mechanisms, while also reducing pathological changes in brain tissue. Meta-analysis, as the highest level of evidence in evidence-based medicine, provides crucial guidance in selecting clinical treatment methods. This paper, through further analysis of collected literature, aims to determine the extent to which acupuncture improves outcomes in the preclinical acute phase of ICH, providing a reference for further research. |  |
| Objectives | 4 | This study aims to systematically evaluate the effectiveness of acupuncture treatment in improving experimental intracerebral hemorrhage outcomes. |  |
| **METHODS** | | |  |
| Eligibility criteria | 5 | The inclusion criteria are as follows: (1) Published animal experiments on ICH with language in either Chinese or English; (2) Intervention group receiving acupuncture or electroacupuncture treatment, control group receiving sham acupuncture or no treatment; (3) Outcome measures include mNSS, Bederson score, Longa score, or BWC. The exclusion criteria are as follows: (1) Unable to extract valid outcome measures; (2) Duplicate publications; (3) Unable to access the full text; (4) Interventions combined with other therapies such as moxibustion, embedded thread at acupoints, etc.; (5) Literature not peer-reviewed, such as conference papers, master's or doctoral theses, or monographs, etc.  Analysis was conducted on four outcome measures: mNSS, Bederson, Longa, and BWC. The samples were divided into acupuncture and control groups for comparison. In studies employing multiple acupuncture methods, the most effective approach was chosen for the acupuncture group. |  |
| Information sources | 6 | Database retrieval includes PubMed, EMBASE, Cochrane Library, Web of Science, Chinese National Knowledge Infrastructure (CNKI), Wanfang Data Information Site, VIP Information Database, and Chinese Biomedical Literature Database (CBM). The time span for retrieval extends from the establishment of the databases to June 2023. |  |
| Search strategy | 7 | Present the full search strategies for all databases, registers and websites, including any filters and limits used. |  |
| Selection process | 8 | Two authors independently screened titles and abstracts from the search records, assessing potentially eligible studies for further evaluation of full texts. In cases of missing information, authors were contacted for clarification. Conflicts were resolved through discussion between the two authors. |  |
| Data collection process | 9 | Two researchers independently extracted data from the included studies, and the data were stored in Excel. Any discrepancies between the two authors were resolved through discussion. Missing information was requested from the original authors via email, and in case of no response, it was noted in the discussion section. |  |
| Data items | 10a | To clarify the therapeutic effect of acupuncture on the acute phase of ICH, data from 3 day of ICH are analyzed. If the experiment involves multiple intervention groups, data extraction was conducted from the group with the most favorable outcomes. |  |
|  | 10b | Extract the following information from the included literature: First author and publication year; Animal gender, species, weight, and sample size (n); Type of anesthetic, injection method, and dosage; Modeling method; Intervention details, including selected acupoints, acupuncture methods, and intervention timing; Control measures; Neurobehavioral and histological indicators (mean ± standard deviation). |  |
| Study risk of bias assessment | 11 | The two researchers independently use SYRCLE's risk of bias tool to assess the quality of the included literature. If there are disagreements during the quality assessment process, these are resolved through discussion or by involving a third researcher. |  |
| Effect measures | 12 | For continuous outcomes, including mNSS, Bederson score, Longa score, and BWC, mean differences and 95% confidence intervals were calculated. |  |
| Synthesis methods | 13a | Tabulating the study intervention characteristics and comparing against the planned groups for each synthesis. |  |
|  | 13b | If the literature presents data in the form of mean ± standard error, we calculate the mean ± standard deviation (SD = SE × √n). If the literature only provides data in image format, we use GetData Graph Digitizer 2.25 to extract the data values from the images. Additionally, we conduct a thorough review of the extracted data, excluding any unreliable data (such as data with excessively small standard deviations, given that neurofunctional scores are typically integers), in order to achieve the most accurate analysis results. |  |
|  | 13c | The study results and assessment of heterogeneity were presented using forest plots. |  |
|  | 13d | We conducted meta-analysis and subgroup analysis using Review Manager 5.3, and performed sensitivity analysis, meta-regression analysis, and publication bias assessment using Stata 14.0. I^2^ was used to evaluate heterogeneity. If I^2^ exceeded 50%, it indicated high heterogeneity and a random-effects model was employed; otherwise, a fixed-effects model was used. |  |
|  | 13e | For outcomes with high heterogeneity, meta-regression analysis was conducted to identify potential sources of heterogeneity. Subgroup analysis for outcome measures with more than ten studies was conducted based on study characteristics, including animal species, ICH modeling methods, acupuncture points, and control measures. |  |
|  | 13f | Sensitivity analysis was performed by systematically excluding individual studies to observe their impact on the overall effect size. |  |
| Reporting bias assessment | 14 | Publication bias assessment was conducted by visualizing a funnel plot. For outcome measures with more than ten studies, Egger's test and the trim-and-fill method were used to assess the degree of bias. |  |
| Certainty assessment | 15 | The two researchers independently use SYRCLE's risk of bias tool to assess the quality of the included literature. |  |
| **RESULTS** | | |  |
| Study selection | 16a | Based on the PRISMA guidelines, we conducted literature screening. Following the search strategy, a total of 1456 articles were retrieved. After removing duplicates, 834 articles remained. Through reviewing titles and abstracts, 633 articles were excluded for the following reasons: (1) Not related to intracerebral hemorrhage; (2) Interventions involving non-acupuncture or combined with other therapies; (3) Human studies; (4) In vitro experiments; (5) Reviews, conference papers, patents, or books; (7) Theses. Among the 202 articles subjected to full-text assessment, 170 were excluded due to inconsistent outcome measures (n=154), unreliable data (n=6), data duplication (n=5), and inability to extract outcome measures (n=5). The remaining 32 studies were included in the meta-analysis. |  |
|  | 16b | 16 were excluded due to unreliable data (n=6), data duplication (n=5), and inability to extract outcome measures (n=5). We conduct a thorough review of the extracted data, excluding any unreliable data (such as data with excessively small standard deviations, given that neurofunctional scores are typically integers), in order to achieve the most accurate analysis results. |  |
| Study characteristics | 17 | Among the 32 included studies, 13 studies utilized Sprague-Dawley (SD) rats, 16 studies used Wistar rats, 2 studies employed guinea pigs, and 1 study utilized spontaneously hypertensive rats (SHR), all of which were male. Regarding anesthesia, 14 studies employed intraperitoneal injection of chloral hydrate, 12 studies used intraperitoneal injection of pentobarbital, and 6 studies did not specify the type of anesthetic used. In terms of modeling, 23 studies induced ICH by autologous blood injection into the caudate nucleus, while 9 studies induced caudate nucleus hemorrhage using collagenase. For the intervention group, 21 studies employed acupuncture from Baihui (GV20) to Qubin (GB7). In the control group, 16 studies performed the same binding fixation procedure as the intervention group but without acupuncture, 15 studies did not implement any measures, and 1 study used sham acupuncture as a control. Regarding outcome measures, 11 studies reported mNSS, 5 studies reported Bederson score, 6 studies reported Longa score, and 14 studies reported BWC. The assessment time ranged from 6 hour to 28 day after ICH surgery, with all studies reporting outcomes on 3 day. |  |
| Risk of bias in studies | 18 | Regarding selection bias, (1) 6 studies (18.75%) used random number table method for grouping, 22 studies (68.75%) did not clearly report the method of random allocation. They only described it as "random" but did not specify the exact method used. 4 studies (12.5%) did not mention "random" or similar content when describing grouping. (2) 1 study (3.13%) reported balanced distribution of baseline characteristics in both intervention and control groups, while 31 studies (96.88%) did not clearly describe baseline characteristics. (3) All studies did not clearly describe allocation concealment, making it impossible to know whether the researchers responsible for acupuncture were aware of the group allocation. For performance bias, (4) 15 studies (46.88%) reported randomization of animal placement, while 17 studies (53.13%) did not clearly mention relevant information. (5) All studies did not mention whether blinding was implemented for caregivers and researchers responsible for acupuncture. For detection bias, (6) 1 study (3.13%) used random number table method during outcome assessment, 2 studies (6.25%) mentioned "random" but did not specify the method used, and 29 studies (90.63%) did not mention "random" or similar content. (7) 1 study (3.13%) implemented blinding for assessors, while 31 studies (96.88%) did not mention the implementation of blinding. For attrition bias, (8) all studies reported complete research data without attrition bias. For reporting bias, (9) 31 studies (96.88%) did not selectively report results, while 1 study (3.13%) had inconsistent descriptions in the methods and results sections, indicating potential selective result reporting. For other bias, (10) 3 studies (9.38%) had other biases. They involved adding new animals to the intervention group or control group to supplement dropouts, but did not describe whether the new animals were subjected to the same low-bias risk operations (such as random allocation and blinding). 29 studies (90.63%) were judged to have no other bias risks. |  |
| Results of individual studies | 19 | In 11 studies, acupuncture demonstrated a significant improvement in mNSS following experimental ICH (n=246, MD=-3.16, 95% CI [-3.66 ~ -2.66], P＜0.00001; heterogeneity Chi2=78.68, df=10 (P＜0.00001), I2=87%,). Among the 5 studies examining Bederson score, acupuncture also showed a significant effect in improving outcomes after experimental ICH (n=98, MD=-0.99, 95% CI [-1.20 ~ -0.78], P＜0.00001; heterogeneity Chi2=4.76, df=4 (P=0.31, I2=16%). Additionally, in 6 studies, acupuncture led to a significant improvement in Longa score after experimental ICH (n=100, MD=-0.54, 95% CI [-0.80 ~ -0.29], P＜0.0001; heterogeneity Chi2=9.91, df=5 (P=0.08), I2=50%). For BWC, 14 studies demonstrated a significant improvement following acupuncture treatment after experimental ICH (n=215, MD=-5.39, 95% CI [-6.90 ~ -3.89], P＜0.00001; heterogeneity Chi2=1180.48, df=13 (P＜0.00001), I2=99%). |  |
| Results of syntheses | 20a | For each synthesis, briefly summarise the characteristics and risk of bias among contributing studies. |  |
|  | 20b | In 11 studies, acupuncture demonstrated a significant improvement in mNSS following experimental ICH (n=246, MD=-3.16, 95% CI [-3.66 ~ -2.66], P＜0.00001; heterogeneity Chi2=78.68, df=10 (P＜0.00001), I2=87%,). Among the 5 studies examining Bederson score, acupuncture also showed a significant effect in improving outcomes after experimental ICH (n=98, MD=-0.99, 95% CI [-1.20 ~ -0.78], P＜0.00001; heterogeneity Chi2=4.76, df=4 (P=0.31, I2=16%). Additionally, in 6 studies, acupuncture led to a significant improvement in Longa score after experimental ICH (n=100, MD=-0.54, 95% CI [-0.80 ~ -0.29], P＜0.0001; heterogeneity Chi2=9.91, df=5 (P=0.08), I2=50%). For BWC, 14 studies demonstrated a significant improvement following acupuncture treatment after experimental ICH (n=215, MD=-5.39, 95% CI [-6.90 ~ -3.89], P＜0.00001; heterogeneity Chi2=1180.48, df=13 (P＜0.00001), I2=99%). |  |
|  | 20c | Due to the observed high heterogeneity in the analysis results for mNSS and BWC (with I2 values of 87% and 99% respectively), we conducted meta-regression analysis to examine the correlation between study characteristics and intervention effects, aiming to identify the sources of this heterogeneity. The results indicate that: (1) for mNSS, the modeling method is a significant source of its heterogeneity (P=0.018 < 0.05); (2) for BWC, acupuncture points (P=0.005 < 0.05) and modeling method (P < 0.001) are significant sources of its heterogeneity. |  |
|  | 20d | The sensitivity analysis results indicate that after removing any individual trial, there were no significant changes in the results for mNSS, Bederson, Longa, and BWC (Figure 3A, 3B, 3C, 3D). This suggests that the stability of the meta-analysis results is good. For both mNSS and BWC outcomes, removing any single study did not lead to significant changes in heterogeneity, indicating that the source of heterogeneity is not attributed to any specific study. |  |
| Reporting biases | 21 | Due to the limited number of studies for both Bederson and Longa (both less than 10), it is not recommended to create a funnel plot. Instead, Egger's test was employed to observe potential publication bias. The funnel plot for mNSS appears to be relatively symmetrical, while the funnel plot for BWC shows some asymmetry. The results of Egger's test confirmed the absence of significant publication bias in mNSS, Bederson, and Longa outcomes, but indicated significant bias in BWC (mNSS P=0.846 > 0.05, Bederson P=0.67 > 0.05, Longa P=0.12 > 0.05, BWC P=0.026 < 0.05). For BWC, we utilized the trim-and-fill method to estimate the number of potentially missing studies and observed changes in the combined effect size estimate. The effect size estimate after correction remained consistent with the uncorrected value (MD -5.39, 95% CI [-6.90 ~ -3.89], P < 0.001), suggesting that there are no "missing" studies. |  |
| Certainty of evidence | 22 | Present assessments of certainty (or confidence) in the body of evidence for each outcome assessed. |  |
| **DISCUSSION** | | |  |
| Discussion | 23a | Li et al. conducted a review of studies up until June 2013, confirming the beneficial effects of scalp acupuncture intervention based on Baihui (GV20) in experimental ICH. However, their review only included literature that incorporated GV20 and is relatively outdated. In our study, we provided the most comprehensive and up-to-date assessment of the effects of acupuncture interventions for experimental ICH. Our findings demonstrate a significant improvement in neurobehavioral and histological outcome measures during the acute phase of experimental ICH, including mNSS (MD=-3.16, P＜0.00001), Bederson score (MD=-0.99, P＜0.00001), Longa score (MD=-0.54, P＜0.0001), and BWC (MD=-5.39, P＜0.00001). |  |
|  | 23b | Firstly, the number of studies on the Bederson and Longa scales is still limited. Secondly, the sample size in animal studies is often small, requiring cautious interpretation of the results. Finally, the methodological quality of the included literature is generally low, especially in Chinese databases, which may pose a potential risk of bias. |  |
|  | 23c | In the subgroup analysis, we only explored the effect estimates of the most commonly used acupoint, Baihui-Qubin (GV20-GB7), compared to other acupoints. |  |
|  | 23d | Acupuncture interventions can significantly improve the neurological and histological outcomes in the acute phase of experimental ICH. Many of the analysis results offer suggestions for clinical ICH trials, such as the selection of acupuncture points and the design of control measures. |  |
| **OTHER INFORMATION** | | |  |
| Registration and protocol | 24a | This study has been registered in PROSPERO under the title: 'Acupuncture for animal models of cerebral hemorrhage: a systematic review and meta-analysis.' Registration number: CRD42023435584. |  |
|  | 24b | A protocol was not prepared. |  |
|  | 24c | Without any amendments to information provided at registration. |  |
| Support | 25 | This work was supported by the National Natural Science Foundation of China [grant number 81774416]. The results of a systematic review do not necessarily represent the views of supporting organizations. |  |
| Competing interests | 26 | The Authors declare that there is no conflict of interest. |  |
| Availability of data, code and other materials | 27 | The data and search strategy for this article is available online. |  |

*From:*  Page MJ, McKenzie JE, Bossuyt PM, Boutron I, Hoffmann TC, Mulrow CD, et al. The PRISMA 2020 statement: an updated guideline for reporting systematic reviews. BMJ 2021;372:n71. doi: 10.1136/bmj.n71

For more information, visit: <http://www.prisma-statement.org/>

### Supplement 2. PRISMA 2020 for Abstracts Checklist

| **Section and Topic** | **Item #** | **Checklist item** | **Reported (Yes/No)** |
| --- | --- | --- | --- |
| **TITLE** | | |  |
| Title | 1 | Systematic Review of Acupuncture Intervention in Experimental Intracerebral Hemorrhage. | Yes |
| **BACKGROUND** | | |  |
| Objectives | 2 | This study aims to systematically evaluate the effectiveness of acupuncture treatment in improving experimental intracerebral hemorrhage outcomes. | Yes |
| **METHODS** | | |  |
| Eligibility criteria | 3 | The inclusion criteria are as follows: (1) Published animal experiments on ICH with language in either Chinese or English; (2) Intervention group receiving acupuncture or electroacupuncture treatment, control group receiving sham acupuncture or no treatment; (3) Outcome measures include mNSS, Bederson score, Longa score, or BWC. The exclusion criteria are as follows: (1) Unable to extract valid outcome measures; (2) Duplicate publications; (3) Unable to access the full text; (4) Interventions combined with other therapies such as moxibustion, embedded thread at acupoints, etc.; (5) Literature not peer-reviewed, such as conference papers, master's or doctoral theses, or monographs, etc. | Yes |
| Information sources | 4 | Database retrieval includes PubMed, EMBASE, Cochrane Library, Web of Science, Chinese National Knowledge Infrastructure (CNKI), Wanfang Data Information Site, VIP Information Database, and Chinese Biomedical Literature Database (CBM). The time span for retrieval extends from the establishment of the databases to June 2023. | Yes |
| Risk of bias | 5 | The two researchers independently use SYRCLE's risk of bias tool to assess the quality of the included literature. If there are disagreements during the quality assessment process, these are resolved through discussion or by involving a third researcher.  Publication bias assessment was conducted by visualizing a funnel plot. For outcome measures with more than ten studies, Egger's test and the trim-and-fill method were used to assess the degree of bias. | Yes |
| Synthesis of results | 6 | Mean Difference (MD), 95% confidence intervals (CI), and P-values were calculated to assess the intergroup differences between the intervention and control groups. I^2^ was used to evaluate heterogeneity. If I^2^ exceeded 50%, it indicated high heterogeneity and a random-effects model was employed; otherwise, a fixed-effects model was used. | Yes |
| **RESULTS** | | |  |
| Included studies | 7 | Included 32 studies with a total sample size of 659. 13 studies utilized Sprague-Dawley (SD) rats, 16 studies used Wistar rats, 2 studies employed guinea pigs, and 1 study utilized spontaneously hypertensive rats (SHR), all of which were male. Regarding anesthesia, 14 studies employed intraperitoneal injection of chloral hydrate, 12 studies used intraperitoneal injection of pentobarbital, and 6 studies did not specify the type of anesthetic used. In terms of modeling, 23 studies induced ICH by autologous blood injection into the caudate nucleus, while 9 studies induced caudate nucleus hemorrhage using collagenase. For the intervention group, 21 studies employed acupuncture from Baihui (GV20) to Qubin (GB7). In the control group, 16 studies performed the same binding fixation procedure as the intervention group but without acupuncture, 15 studies did not implement any measures, and 1 study used sham acupuncture as a control. Regarding outcome measures, 11 studies reported mNSS, 5 studies reported Bederson score, 6 studies reported Longa score, and 14 studies reported BWC. The assessment time ranged from 6 hour to 28 day after ICH surgery, with all studies reporting outcomes on 3 day. | Yes |
| Synthesis of results | 8 | In 11 studies, acupuncture demonstrated a significant improvement in mNSS following experimental ICH (n=246, MD=-3.16, 95% CI [-3.66 ~ -2.66], P＜0.00001; heterogeneity Chi2=78.68, df=10 (P＜0.00001), I2=87%). Among the 5 studies examining Bederson score, acupuncture also showed a significant effect in improving outcomes after experimental ICH (n=98, MD=-0.99, 95% CI [-1.20 ~ -0.78], P＜0.00001; heterogeneity Chi2=4.76, df=4 (P=0.31, I2=16%). Additionally, in 6 studies, acupuncture led to a significant improvement in Longa score after experimental ICH (n=100, MD=-0.54, 95% CI [-0.80 ~ -0.29], P＜0.0001; heterogeneity Chi2=9.91, df=5 (P=0.08), I2=50%). For BWC, 14 studies demonstrated a significant improvement following acupuncture treatment after experimental ICH (n=215, MD=-5.39, 95% CI [-6.90 ~ -3.89], P＜0.00001; heterogeneity Chi2=1180.48, df=13 (P＜0.00001), I2=99%). | Yes |
| **DISCUSSION** | | |  |
| Limitations of evidence | 9 | Firstly, the number of studies on the Bederson and Longa scales is still limited. Secondly, the sample size in animal studies is often small, requiring cautious interpretation of the results. Finally, the methodological quality of the included literature is generally low, especially in Chinese databases, which may pose a potential risk of bias. | Yes |
| Interpretation | 10 | Acupuncture interventions can significantly improve the neurological and histological outcomes in the acute phase of experimental ICH. Many of the analysis results offer suggestions for clinical ICH trials, such as the selection of acupuncture points and the design of control measures. | Yes |
| **OTHER** | | |  |
| Funding | 11 | This work was supported by the National Natural Science Foundation of China [grant number 81774416]. The results of a systematic review do not necessarily represent the views of supporting organizations. | Yes |
| Registration | 12 | This study has been registered in PROSPERO under the title: 'Acupuncture for animal models of cerebral hemorrhage: a systematic review and meta-analysis.' Registration number: CRD42023435584. | Yes |

*From:*  Page MJ, McKenzie JE, Bossuyt PM, Boutron I, Hoffmann TC, Mulrow CD, et al. The PRISMA 2020 statement: an updated guideline for reporting systematic reviews. BMJ 2021;372:n71. doi: 10.1136/bmj.n71

For more information, visit: <http://www.prisma-statement.org/>

### Supplement 3. Search Strategy

**PubMed**

((“Cerebral Hemorrhage”[MeSH]) OR (Hemorrhage, Cerebrum[Title/Abstract]) OR (Cerebrum Hemorrhage[Title/Abstract]) OR (Cerebrum Hemorrhages[Title/Abstract]) OR (Hemorrhages, Cerebrum[Title/Abstract]) OR (Cerebral Parenchymal Hemorrhage[Title/Abstract]) OR (Cerebral Parenchymal Hemorrhages[Title/Abstract]) OR (Hemorrhage, Cerebral Parenchymal[Title/Abstract]) OR (Hemorrhages, Cerebral Parenchymal[Title/Abstract]) OR (Parenchymal Hemorrhage, Cerebral[Title/Abstract]) OR (Parenchymal Hemorrhages, Cerebral[Title/Abstract]) OR (Intracerebral Hemorrhage[Title/Abstract]) OR (Hemorrhage, Intracerebral[Title/Abstract]) OR (Hemorrhages, Intracerebral[Title/Abstract]) OR (Intracerebral Hemorrhages[Title/Abstract]) OR (Hemorrhage, Cerebral[Title/Abstract]) OR (Cerebral Hemorrhages[Title/Abstract]) OR (Hemorrhages, Cerebral[Title/Abstract]) OR (Brain Hemorrhage, Cerebral[Title/Abstract]) OR (Brain Hemorrhages, Cerebral[Title/Abstract]) OR (Cerebral Brain Hemorrhage[Title/Abstract]) OR (Cerebral Brain Hemorrhages[Title/Abstract]) OR (Hemorrhage, Cerebral Brain[Title/Abstract]) OR (Hemorrhages, Cerebral Brain[Title/Abstract])) AND ((“Acupuncture”[MeSH]) OR (“Electroacupuncture”[MeSH]) OR (“Acupuncture Therapy”[MeSH]) OR (“Acupuncture Points”[MeSH]) OR (Pharmacopuncture[Title/Abstract]) OR (Acupuncture Treatment[Title/Abstract]) OR (Acupuncture Treatments[Title/Abstract]) OR (Treatment, Acupuncture[Title/Abstract]) OR (Therapy, Acupuncture[Title/Abstract]) OR (Pharmacoacupuncture Treatment[Title/Abstract]) OR (Treatment, Pharmacoacupuncture[Title/Abstract]) OR (Pharmacoacupuncture Therapy[Title/Abstract]) OR (Therapy, Pharmacoacupuncture[Title/Abstract]) OR (Acupotomy[Title/Abstract]) OR (Acupotomies[Title/Abstract]) OR (Acupuncture Point[Title/Abstract]) OR (Point, Acupuncture[Title/Abstract]) OR (Points, Acupuncture[Title/Abstract]) OR (Acupoints[Title/Abstract]) OR (Acupoint[Title/Abstract])) AND ((“Rats”[MeSH]) OR (“Mice”[MeSH]) OR (“Models, Animal”[MeSH]) OR (“Animal Experimentation”[MeSH]) OR (Rat[Title/Abstract]) OR (Rattus[Title/Abstract]) OR (Rattus norvegicus[Title/Abstract]) OR (Rats, Norway[Title/Abstract]) OR (Rats, Laboratory[Title/Abstract]) OR (Laboratory Rat[Title/Abstract]) OR (Laboratory Rats[Title/Abstract]) OR (Rat, Laboratory[Title/Abstract]) OR (Mus[Title/Abstract]) OR (Mouse[Title/Abstract]) OR (Mus domesticus[Title/Abstract]) OR (Mus musculus domesticus[Title/Abstract]) OR (domesticus, Mus musculus[Title/Abstract]) OR (Mus musculus[Title/Abstract]) OR (Mice, House[Title/Abstract]) OR (House Mice[Title/Abstract]) OR (Mouse, House[Title/Abstract]) OR (House Mouse[Title/Abstract]) OR (Mouse, Swiss[Title/Abstract]) OR (Swiss Mouse[Title/Abstract]) OR (Swiss Mice[Title/Abstract]) OR (Mice, Swiss[Title/Abstract]) OR (Mice, Laboratory[Title/Abstract]) OR (Laboratory Mice[Title/Abstract]) OR (Mouse, Laboratory[Title/Abstract]) OR (Laboratory Mouse[Title/Abstract]) OR (Animal Model[Title/Abstract]) OR (Animal Models[Title/Abstract]) OR (Model, Animal[Title/Abstract]) OR (Laboratory Animal Models[Title/Abstract]) OR (Animal Model, Laboratory[Title/Abstract]) OR (Animal Models, Laboratory[Title/Abstract]) OR (Laboratory Animal Model[Title/Abstract]) OR (Model, Laboratory Animal[Title/Abstract]) OR (Models, Laboratory Animal[Title/Abstract]) OR (Experimental Animal Models[Title/Abstract]) OR (Animal Model, Experimental[Title/Abstract]) OR (Animal Models, Experimental[Title/Abstract]) OR (Experimental Animal Model[Title/Abstract]) OR (Model, Experimental Animal[Title/Abstract]) OR (Models, Experimental Animal[Title/Abstract]) OR (Experimentation, Animal[Title/Abstract]) OR (Animal Research[Title/Abstract]) OR (Research, Animal[Title/Abstract]) OR (Animal Experimental Use[Title/Abstract]) OR (Animal Experimental Uses[Title/Abstract]) OR (Experimental Use, Animal[Title/Abstract]) OR (Experimental Uses, Animal[Title/Abstract]) OR (Animal Experiments[Title/Abstract]) OR (Animal Experiment[Title/Abstract]) OR (Experiment, Animal[Title/Abstract]) OR (Experiments, Animal[Title/Abstract]))

**Embase**

('brain hemorrhage'/exp OR 'brain hemorrhage' OR 'hemorrhage, cerebrum':ab,ti OR 'cerebrum hemorrhage':ab,ti OR 'cerebrum hemorrhages':ab,ti OR 'hemorrhages, cerebrum':ab,ti OR 'cerebral parenchymal hemorrhage':ab,ti OR 'cerebral parenchymal hemorrhages':ab,ti OR 'hemorrhage, cerebral parenchymal':ab,ti OR 'hemorrhages, cerebral parenchymal':ab,ti OR 'parenchymal hemorrhage, cerebral':ab,ti OR 'parenchymal hemorrhages, cerebral':ab,ti OR 'intracerebral hemorrhage':ab,ti OR 'hemorrhage, intracerebral':ab,ti OR 'hemorrhages, intracerebral':ab,ti OR 'intracerebral hemorrhages':ab,ti OR 'hemorrhage, cerebral':ab,ti OR 'cerebral hemorrhages':ab,ti OR 'hemorrhages, cerebral':ab,ti OR 'brain hemorrhage, cerebral':ab,ti OR 'brain hemorrhages, cerebral':ab,ti OR 'cerebral brain hemorrhage':ab,ti OR 'cerebral brain hemorrhages':ab,ti OR 'hemorrhage, cerebral brain':ab,ti OR 'hemorrhages, cerebral brain':ab,ti) AND ('acupuncture'/exp OR 'acupuncture' OR ‘acupuncture point'/exp OR 'acupuncture point' OR 'electroacupuncture'/exp OR 'electroacupuncture' OR 'acupuncture therapy':ab,ti OR pharmacopuncture:ab,ti OR 'acupuncture treatment':ab,ti OR 'acupuncture treatments':ab,ti OR 'treatment, acupuncture':ab,ti OR 'therapy, acupuncture':ab,ti OR 'pharmacoacupuncture treatment':ab,ti OR 'treatment, pharmacoacupuncture':ab,ti OR 'pharmacoacupuncture therapy':ab,ti OR 'therapy, pharmacoacupuncture':ab,ti OR acupotomy:ab,ti OR acupotomies:ab,ti OR 'acupuncture point':ab,ti OR 'point, acupuncture':ab,ti OR 'points, acupuncture':ab,ti OR acupoints:ab,ti OR acupoint:ab,ti) AND ('rat'/exp OR 'rat' OR 'mouse'/exp OR 'mouse' OR 'animal model'/exp OR 'animal model' OR 'animal experiment'/exp OR 'animal experiment' OR ‘Rats’:ab,ti OR ‘Rattus’:ab,ti OR ‘Rattus norvegicus’:ab,ti OR ‘Rats, Norway’:ab,ti OR ‘Rats, Laboratory’:ab,ti OR ‘Laboratory Rat’:ab,ti OR ‘Laboratory Rats’:ab,ti OR ‘Rat, Laboratory’:ab,ti OR ‘Mice’:ab,ti OR ‘Mus’:ab,ti OR ‘Mus domesticus’:ab,ti OR ‘Mus musculus domesticus’:ab,ti OR ‘domesticus, Mus musculus’:ab,ti OR ‘Mus musculus’:ab,ti OR ‘Mice, House’:ab,ti OR ‘House Mice’:ab,ti OR ‘Mouse, House’:ab,ti OR ‘House Mouse’:ab,ti OR ‘Mouse, Swiss’:ab,ti OR ‘Swiss Mouse’:ab,ti OR ‘Swiss Mice’:ab,ti OR ‘Mice, Swiss’:ab,ti OR ‘Mice, Laboratory’:ab,ti OR ‘Laboratory Mice’:ab,ti OR ‘Mouse, Laboratory’:ab,ti OR ‘Laboratory Mouse’:ab,ti OR ‘Models, Animal’:ab,ti OR ‘Animal Models’:ab,ti OR ‘Model, Animal’:ab,ti OR ‘Laboratory Animal Models’:ab,ti OR ‘Animal Model, Laboratory’:ab,ti OR ‘Animal Models, Laboratory’:ab,ti OR ‘Laboratory Animal Model’:ab,ti OR ‘Model, Laboratory Animal’:ab,ti OR ‘Models, Laboratory Animal’:ab,ti OR ‘Experimental Animal Models’:ab,ti OR ‘Animal Model, Experimental’:ab,ti OR ‘Animal Models, Experimental’:ab,ti OR ‘Experimental Animal Model’:ab,ti OR ‘Model, Experimental Animal’:ab,ti OR ‘Models, Experimental Animal’:ab,ti OR ‘Animal Experimentation’:ab,ti OR ‘Experimentation, Animal’:ab,ti OR ‘Animal Research’:ab,ti OR ‘Research, Animal’:ab,ti OR ‘Animal Experimental Use’:ab,ti OR ‘Animal Experimental Uses’:ab,ti OR ‘Experimental Use, Animal’:ab,ti OR ‘Experimental Uses, Animal’:ab,ti OR ‘Animal Experiments’:ab,ti OR ‘Experiment, Animal’:ab,ti OR ‘Experiments, Animal’:ab,ti)

**Cochrane**

ID Search Hits

#1 MeSH descriptor: [Cerebral Hemorrhage] explode all trees 1368

#2 (Hemorrhage, Cerebrum OR Cerebrum Hemorrhage OR Cerebrum Hemorrhages OR Hemorrhages, Cerebrum OR Cerebral Parenchymal Hemorrhage OR Cerebral Parenchymal Hemorrhages OR Hemorrhage, Cerebral Parenchymal OR Hemorrhages, Cerebral Parenchymal OR Parenchymal Hemorrhage, Cerebral OR Parenchymal Hemorrhages, Cerebral OR Intracerebral Hemorrhage OR Hemorrhage, Intracerebral OR Hemorrhages, Intracerebral OR Intracerebral Hemorrhages OR Hemorrhage, Cerebral OR Cerebral Hemorrhages OR Hemorrhages, Cerebral OR Brain Hemorrhage, Cerebral OR Brain Hemorrhages, Cerebral OR Cerebral Brain Hemorrhage OR Cerebral Brain Hemorrhages OR Hemorrhage, Cerebral Brain OR Hemorrhages, Cerebral Brain):ti,ab,kw (Word variations have been searched) 6975

#3 MeSH descriptor: [Acupuncture] explode all trees 713

#4 MeSH descriptor: [Acupuncture Therapy] explode all trees 6405

#5 MeSH descriptor: [Acupuncture Points] explode all trees 2500

#6 MeSH descriptor: [Electroacupuncture] explode all trees 1156

#7 (Pharmacopuncture OR Acupuncture Treatment OR Acupuncture Treatments OR Treatment, Acupuncture OR Therapy, Acupuncture OR Pharmacoacupuncture Treatment OR Treatment, Pharmacoacupuncture OR Pharmacoacupuncture Therapy OR Therapy, Pharmacoacupuncture OR Acupotomy OR Acupotomies OR Acupuncture Point OR Point, Acupuncture OR Points, Acupuncture OR Acupoints OR Acupoint):ti,ab,kw (Word variations have been searched) 17978

#8 MeSH descriptor: [Rats] explode all trees 1459

#9 MeSH descriptor: [Mice] explode all trees 1721

#10 MeSH descriptor: [Models, Animal] explode all trees 911

#11 MeSH descriptor: [Animal Experimentation] explode all trees 9

#12 (Rat OR Rattus OR Rattus norvegicus OR Rats, Norway OR Rats, Laboratory OR Laboratory Rat OR Laboratory Rats OR Rat, Laboratory OR Mus OR Mouse OR Mus domesticus OR Mus musculus domesticus OR domesticus, Mus musculus OR Mus musculus OR Mice, House OR House Mice OR Mouse, House OR House Mouse OR Mouse, Swiss OR Swiss Mouse OR Swiss Mice OR Mice, Swiss OR Mice, Laboratory OR Laboratory Mice OR Mouse, Laboratory OR Laboratory Mouse OR Animal Model OR Animal Models OR Model, Animal OR Laboratory Animal Models OR Animal Model, Laboratory OR Animal Models, Laboratory OR Laboratory Animal Model OR Model, Laboratory Animal OR Models, Laboratory Animal OR Experimental Animal Models OR Animal Model, Experimental OR Animal Models, Experimental OR Experimental Animal Model OR Model, Experimental Animal OR Models, Experimental Animal OR Experimentation, Animal OR Animal Research OR Research, Animal OR Animal Experimental Use OR Animal Experimental Uses OR Experimental Use, Animal OR Experimental Uses, Animal OR Animal Experiments OR Animal Experiment OR Experiment, Animal OR Experiments, Animal):ti,ab,kw (Word variations have been searched) 28156

#13 #1 OR #2 6984

#14 #3 OR #4 OR #5 OR #6 OR #7 18371

#15 #8 OR #9 OR #10 OR #11 OR #12 28165

#16 #13 AND #14 AND #15 2

**Web of Science**

TS=(Cerebral Hemorrhage OR Hemorrhage, Cerebrum OR Cerebrum Hemorrhage OR Cerebrum Hemorrhages OR Hemorrhages, Cerebrum OR Cerebral Parenchymal Hemorrhage OR Cerebral Parenchymal Hemorrhages OR Hemorrhage, Cerebral Parenchymal OR Hemorrhages, Cerebral Parenchymal OR Parenchymal Hemorrhage, Cerebral OR Parenchymal Hemorrhages, Cerebral OR Intracerebral Hemorrhage OR Hemorrhage, Intracerebral OR Hemorrhages, Intracerebral OR Intracerebral Hemorrhages OR Hemorrhage, Cerebral OR Cerebral Hemorrhages OR Hemorrhages, Cerebral OR Brain Hemorrhage, Cerebral OR Brain Hemorrhages, Cerebral OR Cerebral Brain Hemorrhage OR Cerebral Brain Hemorrhages OR Hemorrhage, Cerebral Brain OR Hemorrhages, Cerebral Brain) AND TS=(Acupuncture OR Acupuncture Therapy OR Acupuncture Points OR Electroacupuncture OR Pharmacopuncture OR Acupuncture Treatment OR Acupuncture Treatments OR Treatment, Acupuncture OR Therapy, Acupuncture OR Pharmacoacupuncture Treatment OR Treatment, Pharmacoacupuncture OR Pharmacoacupuncture Therapy OR Therapy, Pharmacoacupuncture OR Acupotomy OR Acupotomies OR Acupuncture Point OR Point, Acupuncture OR Points, Acupuncture OR Acupoints OR Acupoint) AND TS=(Rats OR Rat OR Rattus OR Rattus norvegicus OR Rats, Norway OR Rats, Laboratory OR Laboratory Rat OR Laboratory Rats OR Rat, Laboratory OR Mice OR Mus OR Mouse OR Mus domesticus OR Mus musculus domesticus OR domesticus, Mus musculus OR Mus musculus OR Mice, House OR House Mice OR Mouse, House OR House Mouse OR Mouse, Swiss OR Swiss Mouse OR Swiss Mice OR Mice, Swiss OR Mice, Laboratory OR Laboratory Mice OR Mouse, Laboratory OR Laboratory Mouse OR Models, Animal OR Animal Model OR Animal Models OR Model, Animal OR Laboratory Animal Models OR Animal Model, Laboratory OR Animal Models, Laboratory OR Laboratory Animal Model OR Model, Laboratory Animal OR Models, Laboratory Animal OR Experimental Animal Models OR Animal Model, Experimental OR Animal Models, Experimental OR Experimental Animal Model OR Model, Experimental Animal OR Models, Experimental Animal OR Animal Experimentation OR Experimentation, Animal OR Animal Research OR Research, Animal OR Animal Experimental Use OR Animal Experimental Uses OR Experimental Use, Animal OR Experimental Uses, Animal OR Animal Experiments OR Animal Experiment OR Experiment, Animal OR Experiments, Animal)

**CNKI**

（主题：脑出血 + 自发性脑出血 + 原发性脑出血 + 出血性脑卒中 + 出血中风 + 脑充血 + 脑溢血（精确））AND（主题：针刺 + 普通针刺 + 毫针针刺 + 毫针刺 + 体针针刺 + 电针 + 声波电针 + 头部电针 + 脉冲电针 + 针刺疗法（精确））AND（主题：大鼠 + SD + SD大鼠 + 鼠类 + 啮齿动物 + 小鼠 + 啮齿类动物 + 动物 + 实验 + 模型 + 动物实验 + 实验动物 + 动物模型 + 实验动物模型 + 动物研究（精确））

**WANFANG**

主题:(脑出血 or 自发性脑出血 or 原发性脑出血 or 出血性脑卒中 or 出血中风 or 脑溢血 or 脑充血) and 主题:(针刺 or 普通针刺 or 毫针针刺 or 毫针刺 or 体针针刺 or 电针 or 头部电针 or 声波电针 or 脉冲电针 or 针刺疗法) and 主题:(大鼠 or SD or SD大鼠 or 鼠类 or 啮齿动物 or 小鼠 or 啮齿类动物 or 动物 or 实验 or 模型 or 动物实验 or 实验动物 or 动物模型 or 实验动物模型 or 动物研究)

**VIP**

((((((((题名或关键词=脑出血 OR 题名或关键词=自发性脑出血) OR 题名或关键词=原发性脑出血) OR 题名或关键词=出血性脑卒中) OR 题名或关键词=出血中风) OR 题名或关键词=脑溢血) OR 题名或关键词=脑充血) AND (((((((((题名或关键词=针刺 OR 题名或关键词=普通针刺) OR 题名或关键词=毫针针刺) OR 题名或关键词=毫针刺) OR 题名或关键词=体针针刺) OR 题名或关键词=电针) OR 题名或关键词=头部电针) OR 题名或关键词=声波电针) OR 题名或关键词=脉冲电针) OR 题名或关键词=针刺疗法)) AND ((((((((((((((题名或关键词=大鼠 OR 题名或关键词=SD) OR 题名或关键词=SD大鼠) OR 题名或关键词=鼠类) OR 题名或关键词=啮齿动物) OR 题名或关键词=小鼠) OR 题名或关键词=啮齿类动物) OR 题名或关键词=动物) OR 题名或关键词=实验) OR 题名或关键词=模型) OR 题名或关键词=动物实验) OR 题名或关键词=实验动物) OR 题名或关键词=动物模型) OR 题名或关键词=实验动物模型) OR 题名或关键词=动物研究))

**CBM**

("脑出血"[不加权:扩展] OR "自发性脑出血"[常用字段:智能] OR "原发性脑出血"[常用字段:智能] OR "出血性脑卒中"[常用字段:智能] OR "出血中风"[常用字段:智能] OR "脑溢血"[常用字段:智能] OR "脑充血"[常用字段:智能]) AND ("针刺疗法"[不加权:扩展] OR "电针"[不加权:扩展] OR "针刺"[常用字段:智能] OR "普通针刺"[常用字段:智能] OR "毫针针刺"[常用字段:智能] OR "毫针刺"[常用字段:智能] OR "体针针刺"[常用字段:智能] OR "头部电针"[常用字段:智能] OR "声波电针"[常用字段:智能] OR "脉冲电针"[常用字段:智能]) AND ("大鼠, Sprague-Dawley"[不加权:扩展] OR "大鼠, Wistar"[不加权:扩展] OR "小鼠, 裸"[不加权:扩展] OR "小鼠, 近交BALB C"[不加权:扩展] OR "小鼠, 近交C57BL"[不加权:扩展] OR "模型, 动物"[不加权:扩展] OR "动物实验"[不加权:扩展] OR "大鼠"[常用字段:智能] OR "SD"[常用字段:智能] OR "SD大鼠"[常用字段:智能] OR "鼠类"[常用字段:智能] OR "啮齿动物"[常用字段:智能] OR "小鼠"[常用字段:智能] OR "啮齿类动物"[常用字段:智能] OR "动物"[常用字段:智能] OR "实验"[常用字段:智能] OR "模型"[常用字段:智能] OR "实验动物"[常用字段:智能] OR "动物模型"[常用字段:智能] OR "实验动物模型"[常用字段:智能] OR "动物研究"[常用字段:智能])
